# Functional *cis*-regulatory modules encoded by mouse-specific endogenous retrovirus

**DOI:** 10.1038/ncomms14550

**Published:** 2017-03-28

**Authors:** Vasavi Sundaram, Mayank N. K. Choudhary, Erica Pehrsson, Xiaoyun Xing, Christopher Fiore, Manishi Pandey, Brett Maricque, Methma Udawatta, Duc Ngo, Yujie Chen, Asia Paguntalan, Tammy Ray, Ava Hughes, Barak A. Cohen, Ting Wang

**Affiliations:** 1 Division of Biological and Biomedical Sciences, Washington University School of Medicine, 660 S. Euclid Avenue, St. Louis, Missouri 63110, USA; 2 Department of Genetics, Center for Genome Sciences and Systems Biology, Washington University School of Medicine, 4515 McKinley Avenue, St. Louis, Missouri 63110, USA

## Abstract

*Cis*-regulatory modules contain multiple transcription factor (TF)-binding sites and integrate the effects of each TF to control gene expression in specific cellular contexts. Transposable elements (TEs) are uniquely equipped to deposit their regulatory sequences across a genome, which could also contain *cis*-regulatory modules that coordinate the control of multiple genes with the same regulatory logic. We provide the first evidence of mouse-specific TEs that encode a module of TF-binding sites in mouse embryonic stem cells (ESCs). The majority (77%) of the individual TEs tested exhibited enhancer activity in mouse ESCs. By mutating individual TF-binding sites within the TE, we identified a module of TF-binding motifs that cooperatively enhanced gene expression. Interestingly, we also observed the same motif module in the *in silico* constructed ancestral TE that also acted cooperatively to enhance gene expression. Our results suggest that ancestral TE insertions might have brought in *cis*-regulatory modules into the mouse genome.

## Abstract (web summary)

The gene-battery model posits transposable elements (TEs) may be *cis*-regulatory elements to control gene expression. Here, mouse-specific TEs are shown as binding sites for multiple collaborating transcription factors in embryonic stem cells, and act as *cis*-regulatory modules in synergistic fashion.

Transposable elements (TEs) are widely known for their capability to affect gene expression, beginning with Barbara McClintock's seminal maize experiments^1^,^2^. In eukaryotes, TEs are widespread and constitute almost half of the human genome^3^,^4^,^5^ and therefore could be broadly impacting gene regulation. However, TEs were thought to be ‘junk' DNA, and largely ignored from many studies. Over the past decade, numerous studies have robustly shown that TEs could be gene regulatory elements, as they contribute binding sites for many individual transcription factors (TFs), including p53, Nanog, Oct4 and CTCF in various cell types^6^,^7^,^8^,^9^,^10^, and have epigenetic signatures of transcriptional enhancers in these cells^11^,^12^,^13^,^14^.

A few studies have demonstrated that TEs indeed can rewire existing gene regulatory networks, supporting Britten and Davidson's hypothesis^15^,^16^. This hypothesis postulated that repetitive sequences are an efficient evolutionary mechanism for rapidly depositing *cis*-regulatory modules of TF-binding sites and coordinating the transcription of multiple genes at the same developmental stage^15^,^16^,^17^. This hypothesis builds on the ability of TEs to mobilize and repeatedly deposit its sequence across the genome. In addition, it is still unknown if the ancestral TE had transcriptional regulatory sites and the regulatory potential, or if it is acquired later on in evolutionary time^18^. Here we focus on characterizing the gene regulatory potential of TEs by investigating *cis*-regulatory modules encoded in TEs.

An interesting part of the Britten and Davidson hypothesis is the theoretical description of TEs containing not only *cis*-regulatory sites, but also *cis*-regulatory modules. TE-derived modules of TF-binding sites make an interesting model for studying the regulatory role and innovation contributed by TEs to a genome. A TE-derived *cis*-regulatory module represents a collection of TF-binding sites that together regulate the expression of nearby target genes. Although there are no well-characterized cases of TE-derived *cis*-regulatory modules that we know of, this will provide important support to the regulatory innovation contributed by TEs to a genome. The benefit of TE-derived *cis*-regulatory modules is that TEs form an efficient evolutionary mechanism for the rapid spread of *cis*-regulatory modules, as opposed to alternatively acquiring the *cis*-regulatory module via point mutations in the sequence. Here we study and characterize a module of binding sites for pluripotency TFs in mouse embryonic stem cells (ESCs) in conjunction with the associated ability of these TEs to regulate gene expression. We observe that indeed the TE-derived TF-binding modules enhance gene expression synergistically. Interestingly, our results indicate that the TEs likely acquired the potential to regulate gene expression from their ancestral sequences.

## Results

### LTRs enriched for clusters of pluripotency TF-binding sites

**Figure 1:**
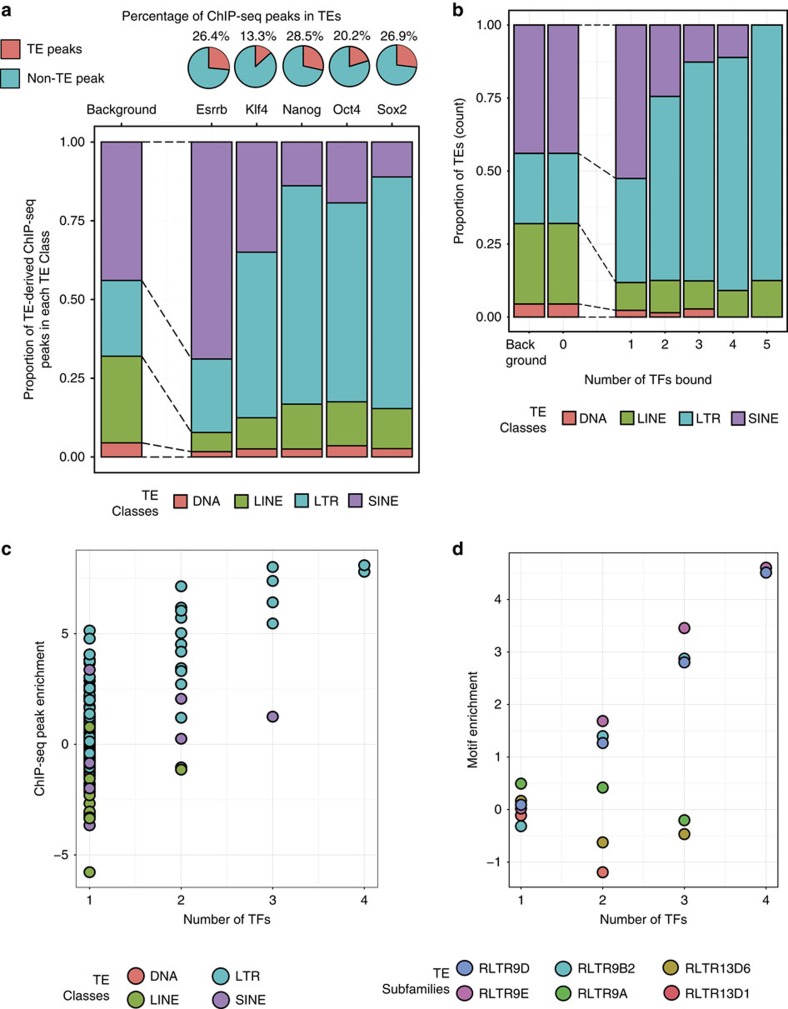
TEs enriched for multiple pluripotency TF-binding sites. (**a**) Upper panel: percentage of ChIP-seq peaks for each of the five pluripotency TFs, in TEs (red pie in chart). Lower panel: proportion of TEs bound by each TF, categorized by TE classes. ‘Background' represents the distribution of TEs in the genome. (**b**) Proportion of TEs bound by one to five pluripotency TFs, categorized by TE classes. ‘Background' represents the distribution of TEs in the genome. (**c**) Left panel: enrichment score (log odd ratio; *y* axis) of various number TFs' ChIP-seq peaks (*x* axis) in individual TE subfamilies (each dot). (**d**) Enrichment (log odds ratio; *y* axis) of clusters of TF-binding motifs in TE subfamilies that were enriched for multiple TF ChIP-seq peaks, categorized by the number of TFs with motifs in the TE (*x* axis).

We used chromatin immunoprecipitation-sequencing (ChIP-seq) to analyse the binding of five pluripotency TFs in mouse ESCs^19^, and identified ChIP-seq peaks overlapping RepeatMasker-annotations of TEs (see Methods; ref. 10). The five TFs represent three master regulators of pluripotency (Nanog, Oct4 and Sox2)^19^,^20^,^21^,^22^,^23^,^24^ and two reprogramming factors (Esrrb and Klf4)^25^,^26^. On average, 23% of the *in vivo* binding sites for these five TFs occur in TEs (Fig. 1a; Supplementary Table 1). The majority of the TE-derived ChIP-seq binding peaks occur in long terminal repeat (LTR) elements, with the exception of Esrrb that has the majority of its TE derived ChIP-seq binding peaks in short interspersed nuclear elements (SINE); both of these observations are in agreement with previous studies^10^,^27^. We then identified TEs bound by multiple TFs by overlapping TEs with genomic regions containing multiple TFs' ChIP-seq peaks (see Methods; Supplementary Fig. 1). Overall, 21% of genomic regions bound by two or more TFs (1,331 out of 6,366), occurred in TEs (Supplementary Table 2). Interestingly, LTRs represent 24% of TEs in the mouse genome, but 70% of TEs bound by two or more TFs occurs in LTR elements (Fig. 1b).

To determine whether specific TE subfamilies contributed these clusters of TF-binding sites, we measured the enrichment (log odds ratio, LOR) of binding sites for various numbers of TFs in each TE subfamily (see Methods). Certain TE subfamilies showed strong enrichment (LOR≥4.5 and hypergeometric *P* value<0.001; Fig. 1c; Supplementary Table 3) for being bound by multiple TFs. We looked at TE subfamilies that were highly enriched for multiple TF-binding sites, and found five TE subfamilies (RLTR9A, RLTR9B2, RLTR9D, RLTR9E and RLTR13D6; all belonging to the ERVK family of TEs in mouse) that had at least 64-fold enrichment (that is, LOR≥6) for clusters of *in vivo* TF binding (Supplementary Table 4). We added RLTR13D1 in this list, which by nomenclature is similar to RLTR13D6 and also showed strong enrichment (Supplementary Table 3; LOR≥1.5; hypergeometric *P* value<0.001) of various individual TF's binding. The six TE subfamilies enriched for multiple TF-binding motifs (see Methods; Fig. 1d). Since genomic copies of a TE subfamily are phylogenetically related and result from the expansion of an element, these subfamilies are candidates for examining the spread of *cis*-regulatory modules by TEs. We focused the rest of our analysis on these six candidate TE subfamilies to determine whether these TE subfamilies are indeed spreading regulatory modules.

### TEs containing multiple TF-binding sites are mouse specific

To determine the evolutionary impact of the six TE subfamilies that enrich for multiple TF-binding sites, we estimated the evolutionary age of these TE subfamilies using three methods. First, we asked in what existing species are these TE sequences present. Using BLAST^28^, we searched across various vertebrate genomes for sequences homologous to the RepBase-consensus sequence^29^ of each of the six TE subfamilies (see Methods; Supplementary Fig. 2). We can reliably detect these TEs only in the mouse genome and not in other vertebrate genomes, including the rat genome.

**Figure 2:**
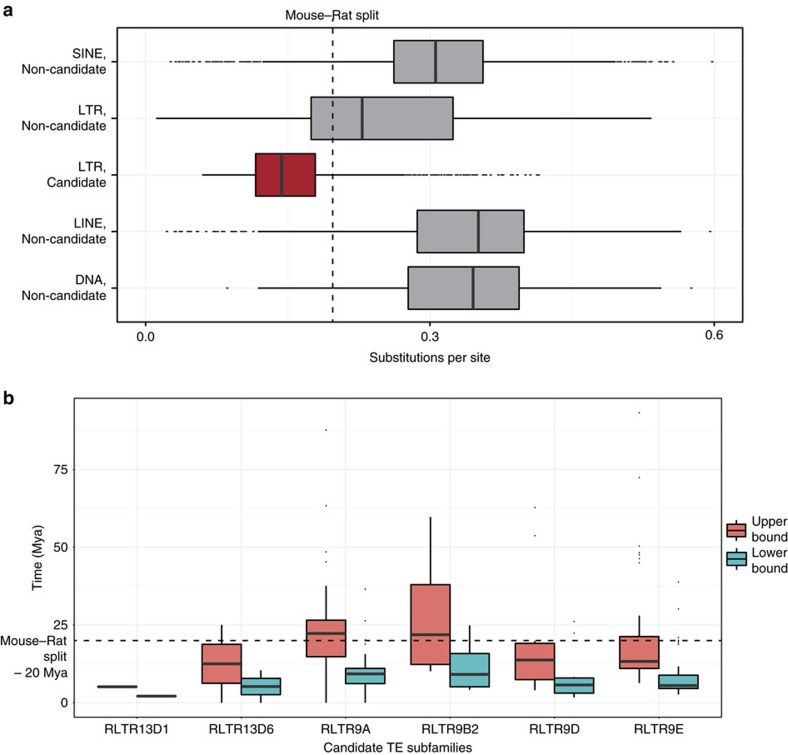
Evolutionary age estimates of TE subfamilies enriched for multiple TF-binding sites. (**a**) Distribution of the substitution rate (that is, substitutions per site), based on the Juke–Cantor model for individual elements. ‘Candidate' represents the six TE subfamilies (that is, RLTR9A, RLTR9B2, RLTR9D, RLTR9E, RLTR13D1 and RLTR13D6), while all other TE subfamilies are labelled ‘Non-candidate'. See Methods for details on calculating the substitution rate. (**b**) Estimates of the time of insertion (*y* axis; million years ago (Mya)) of the six TE subfamilies (*x* axis). See Methods for details on calculation of time of insertion. We used two estimates of mutation rates—an upper and lower bound (as labelled in the figure legend)—to estimate the time of insertion. In box plots, the centre line represents the median, the box limits represent the 25th and 75th percentiles and the whiskers represent the minimum and maximum values in the interquartile range.

Second, we used sequence divergence of TEs to estimate their age. We used the RepBase-consensus sequence^29^ of each TE subfamily as a proxy for the ancestral state of the TE^30^,^31^ and estimated the sequence divergence between the genomic copies of each TE subfamily to its corresponding RepBase-consensus sequence, using the Juke-Cantor model^32^ (see Methods). The evolutionary distance of each element (see Methods) was compared with that of mouse and rat (under the neutral evolutionary model), which is estimated to be 0.15–0.2 per site^5^,^33^. The six TE subfamilies have the smallest evolutionary distance (Fig. 2a), compared with other classes of TE subfamilies. The small evolutionary distance suggests that these six TE subfamilies have not been in the mouse genome for long, and most likely came into existence after the mouse–rat split.

Third, we calculated when these LTR subfamilies entered the mouse genome. At the time of insertion of an endogenous retrovirus (ERV) sequence, the sequence of the two LTRs flanking the ERV is identical, but post insertion, the LTRs evolve independently and their sequence diverges. By estimating the sequence variation between two LTR sequences of a nearly intact ERV sequence, the time of insertion of the element can be calculated^34^ (see Methods). We estimated the time of insertion of the TEs to be <12–14 million years ago, corresponding to after the mouse–rat split (Fig. 2b). Together, these age estimates establish that these six TE subfamilies are mouse-specific.

### Enhancer signature associated with TEs bound by multiple TFs

**Figure 3:**
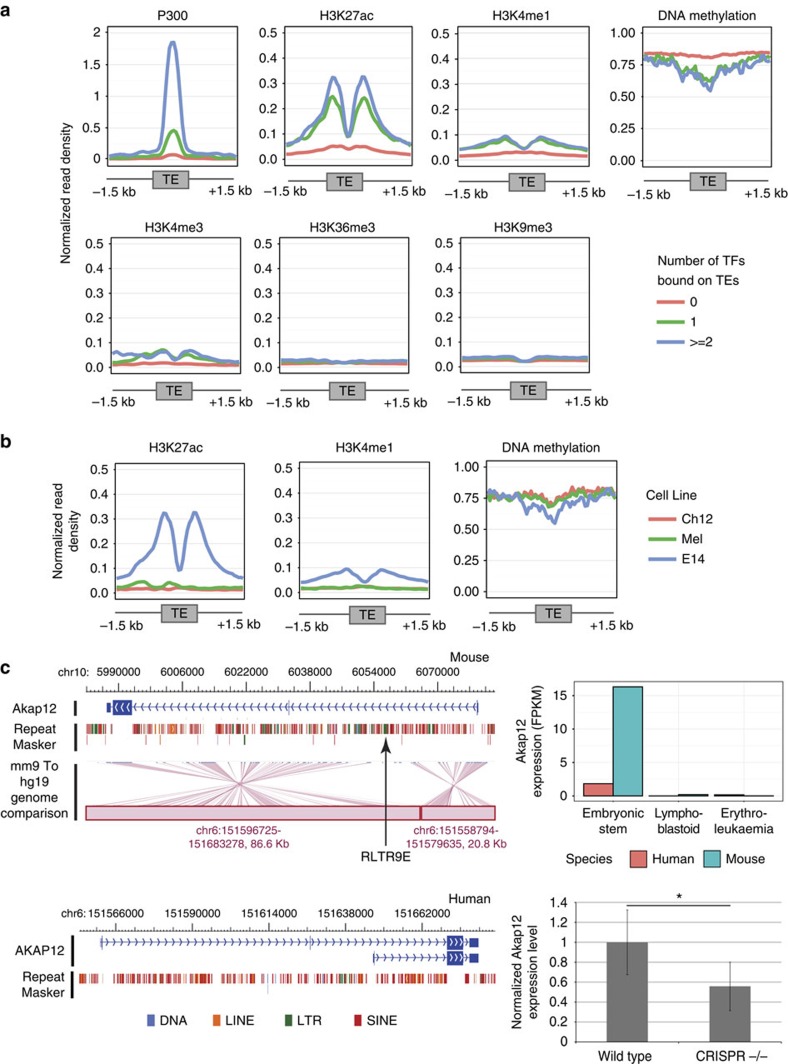
Epigenetic signature of TEs bound by pluripotency TFs. (**a**) Normalized read density of various epigenetic marks in mouse ESCs (E14) on 3-kb regions centred on TEs (belonging to the six candidate TE subfamilies—RLTR9A, RLTR9B2, RLTR9D, RLTR9E, RLTR13D1 and RLTR13D6) that were bound by one and two or more pluripotency TFs, compared to TEs from the same subfamilies that were not bound. (**b**) Normalized read density of enhancer epigenetic marks in mouse ES (E14), lymphoblastoid (Ch12) and erythroleukemia (Mel) cell lines, on 3-kb regions centred on TEs (that is, six candidate TE subfamilies) that were bound by two or more TFs. (**c**) Genome view (WashU Epigenome Browser)^68^ of the *Akap12* locus in the mouse genome (mm9: top-left panel), and human genome (hg19: bottom-left panel). The genome comparison track (mm9 to hg19) indicates orthologous regions in mouse (blue) and human (red). The mouse-specific RLTR9E element is marked in the mouse panel. Top-right panel: expression level (FPKM) of *Akap12* that is likely a target of the RLTR9E element and exhibits a mouse ESC-specific expression pattern (as identified by DESeq analysis—see Methods). Bottom-right panel: normalized expression level of *Akap12* (measured by qRT-PCR) in mouse ESCs (labelled ‘Wild type') and when the RLTR9E element was deleted (using CRISPR/Cas9; labelled ‘CRISPR−/−'). * represents *P* value=0.02, as measured by Student's *t*-test across two biological and three technical replicates. Error bars represent s.d.

Next, we set out to define an epigenetic signature of the TEs bound by multiple TFs (see Methods). As expected, candidate TEs that encoded multiple TF-binding sites were marked by increased H3K4me1 and H3K27ac, bound by P300, and had reduced DNA methylation, which are known signatures of transcriptional enhancers (Fig. 3a). Interestingly, TEs bound by two or more TFs had a relatively stronger enhancer signature (that is, higher normalized read density of P300, H3K4me1 and H3K27ac, and lower DNA methylation signal), than TEs that had one or no TFs bound to it (Fig. 3a). We observed a similar difference in epigenetic signatures of non-TE sequences that were bound by one and two or more TFs (Supplementary Fig. 3A). As expected, the TEs had no signal of H3K36me3 and H3K9me3, and a very low signature of H3K4me3 (ref. 10). Overall, these results indicate that TEs bound by multiple TFs could function as transcriptional enhancers in mouse ESCs.

We then compared the epigenetic signatures of TEs bound by two or more TFs in mouse ESCs, with other mouse cells to determine the cell-type specificity of this enhancer epigenetic signature (see Methods). The enhancer signature of TEs bound by multiple TFs was specific to mouse ESCs and absent in other cell types (Fig. 3b). Correspondingly, the TEs bound by multiple TFs showed increased DNA methylation in the other cell types, suggesting that their transcriptional activity was epigenetically suppressed in other cell types. Non-TE sequences bound by two or more TFs also showed the same cell-type specificity (Supplementary Fig. 3B). Together, these results indicate that TEs bound by multiple TFs could be ESC-specific enhancers.

To determine the effect of TEs bound by multiple TFs on nearby gene expression, we analysed mouse ESC transcriptome data. Since the TEs contain modules of pluripotency TF-binding sites, and are mouse-specific sequences, we expected that the target genes of these elements would be mouse ESC specifically expressed. We identified mouse ESC specifically expressed genes (see Methods; Supplementary Fig. 4) and found that a small fraction of these genes (∼10%,) can be associated with a nearby TE-derived TF-binding module belonging to the six TE subfamilies. Specifically, 10% (39 out of 376) of the TEs are near (that is, within 50 kb) mouse ESC specifically expressed genes, and can be potential regulators of the gene's mouse-specific expression pattern. One such gene is *Akap12* (a kinase anchor protein 12, Fig. 3c) that contains a RLTR9E element bound by four TFs in its first intron (Supplementary Fig. 5). Although there is no known functional role of *Akap12* in mouse ESCs specifically, *Akap12* is thought to be a tumor suppressor gene, and integrally involved in cellular signalling pathways^35^,^36^,^37^,^38^. To test the effect of the RLTR9E element on the expression level of *Akap12* in mouse ESCs, we used CRISPR/Cas9 to delete the RLTR9E element, and subsequently quantified the change in expression of *Akap12* (Fig. 3c). We observed that the deletion of the RLTR9E element resulted in a significant reduction in the expression level of *Akap12* (that is, 45% reduction; Student's *t*-test, *P* value<0.05), and no significant change in nearby genes (Supplementary Fig. 6). This kind of TE-mediated gene regulation might demonstrate one mechanism for creating variation in the signalling pathways between human and mouse pluripotency networks^39^.

### TF-binding motifs in TEs enhance gene expression as a module

**Figure 4:**
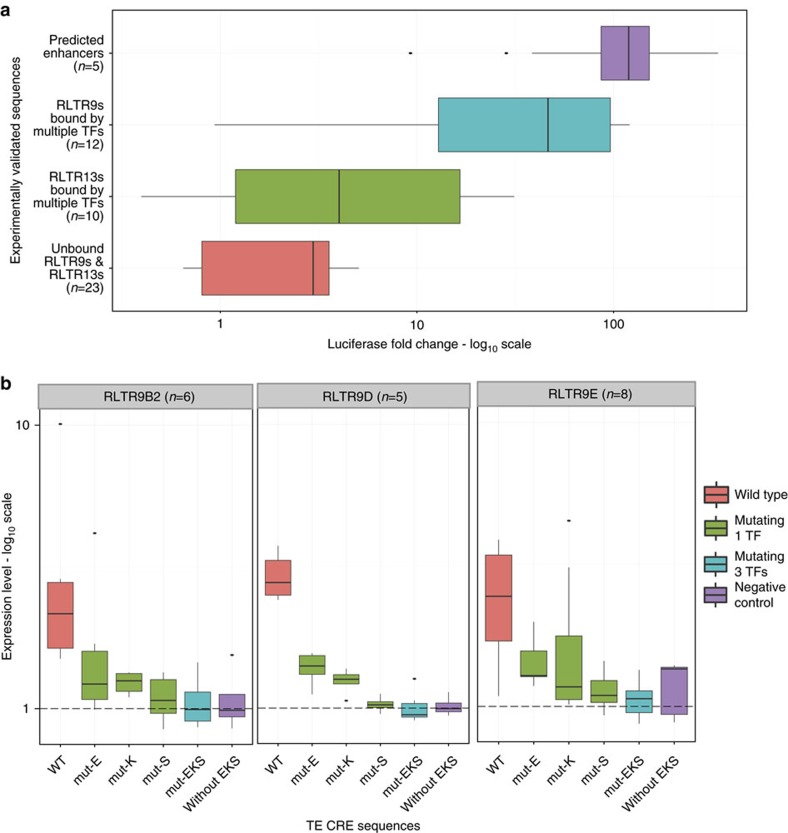
Regulatory potential of TEs bound by multiple TFs in mouse ESCs. (**a**) Luciferase fold change (log_10_ scale) of TEs bound by multiple TFs (RLTR9s and RLTR13s—labelled ‘RLTR9s bound by multiple TFs' and ‘RLTR13s bound by multiple TFs'), compared with TEs from the same subfamilies with comparable length but no motifs (labelled ‘Unbound RLTR9s & RLTR13s'), and non-TE regions bound by multiple TFs (labelled ‘Predicted enhancers'). The average luciferase fold change of each TE from three technical replicates is plotted as a boxplot on a log_10_ scale. (**b**) Expression values (from CRE-seq^67^) for TE CREs containing the EKS motif module and their respective mutant sequences (single mutants labelled ‘Mutating 1 TF' and triple mutants labelled ‘Mutating 3 TFs', in the legend), along with TEs from the same subfamilies lacking the EKS motifs (as a negative control; labelled ‘Negative Control' in the legend). Boxplots representing the expression level of TE CREs is measured by the ratio of the read counts of cDNA to DNA, per barcode. A panel represents each subfamily, and ‘*n*' represents the number of genomic copies that were tested. In box plots, the centre line represents the median, the box limits represent the 25th and 75th percentiles and the whiskers represent the minimum and maximum values in the interquartile range.

To determine whether TEs bound by multiple TFs can regulate gene expression, we experimentally evaluated the enhancer function of 22 TEs (Supplementary Fig. 7; Supplementary Table 5A) in mouse ESCs, using a luciferase assay (see Methods). We selected 22 elements from the six TE subfamilies (12 from the four RLTR9 subfamilies and 10 from the two RLTR13 subfamilies) that contained multiple TF-binding sites. These elements were compared to non-TE genomic regions that also contained multiple TF-binding sites and could be enhancers^40^, along with TEs from the same subfamilies that were of similar length but lacked motifs (Supplementary Tables 5B,C). The TEs had regulatory potentials (defined as the luciferase fold change compared to the basal/empty-vector sequence) that were comparable to non-TE genomic regions that were bound by multiple TFs, and higher than TEs from the same subfamily that lacked multiple TF-binding sites (Fig. 4a). The majority of the TEs (∼77%; 17 out of 22) enhanced luciferase gene expression (luciferase fold change>2), while almost half of them showed strong ability to enhance luciferase expression (luciferase fold change>10, upto 252 fold; Supplementary Fig. 8). Overall, the four RLTR9 subfamilies had higher regulatory potential than the two RLTR13 subfamilies (Fig. 4a), which can be due to differences in the motif content in addition to sequence content (Supplementary Fig. 9). We correlated the regulatory potential of TEs with the number of TF-binding sites, and observed that TEs that were bound by more TFs had higher regulatory potential (Supplementary Fig. 10). With respect to motifs, TEs with three binding motifs resulted in the highest regulatory potential (Supplementary Fig. 10), from our data set. TEs with more or less than three binding motifs had relatively lower regulatory potentials.

Next, we determined the effect of TF-binding sites on the TE's ability to regulate gene expression. We identified that three out of the six TE subfamilies that could regulate gene expression had binding sites for Esrrb, Klf4 and Sox2 (EKS) in the upstream part of their sequence (Supplementary Fig. 9). We sought to characterize the effect of these motifs on the TE's regulatory potential by mutagenesis experiments. First, we used a luciferase reporter assay to test whether the multiple motifs in a TE showed cooperativity in its ability to enhance gene expression (see Methods; Supplementary Fig. 11; Supplementary Tables 6A,B). Indeed, we found in three of the TEs (belonging to RLTR9B2, RLTR9D and RLTR9E subfamilies), the EKS motif module works synergistically to enhance gene expression. In other words, the wild-type luciferase fold change is a result of all three motifs, and is reduced when any one of the motifs are absent. Then, to determine whether this cooperativity between the motifs in the EKS motif module was a feature of the TEs belonging to these three subfamilies, we selected 19 elements from these three subfamilies (Supplementary Fig. 12; Supplementary Tables 7 and 8) and used *cis*-regulatory element-sequencing (CRE-seq)^67^, which is a high-throughput reporter assay to characterize the effect of the motifs on the TE's regulatory potential (see Methods).

We identified that mutations to the EKS motifs reduce the expression of the wild-type TE sequence (Fig. 4b). Mutating any one of the EKS motifs resulted in a large decrease in the regulatory potential of the element (Student's *t*-test *P* value<0.05). Although mutating any one motif reduced the regulatory potential of the wild-type sequence, the extent to which the mutation reduced the regulatory potential was different for different motifs and TE sequences. Importantly, the reduction in regulatory potential caused by mutating one motif is comparable to mutating all three motifs. Our results show that the effect of the motifs in the EKS cluster is greater than the sum of each motif. Since the regulatory potential of the TEs is dependent on the presence of all three motifs, it suggests that this EKS motif module in TEs work cooperatively to enhance gene expression. We also observed that mutating all motifs in the EKS motif module reduced the expression of the wild-type sequence and made it comparable to TEs from the same subfamily (that is, similar sequences) that lacked the EKS motifs (Student's *t*-test *P* value>0.05). This suggests that the EKS motif module drives the regulatory potential observed from these TEs, and more importantly the EKS motifs work as a module that synergistically enhance gene expression.

### The ancestral state of TEs also enhance gene expression

**Figure 5:**
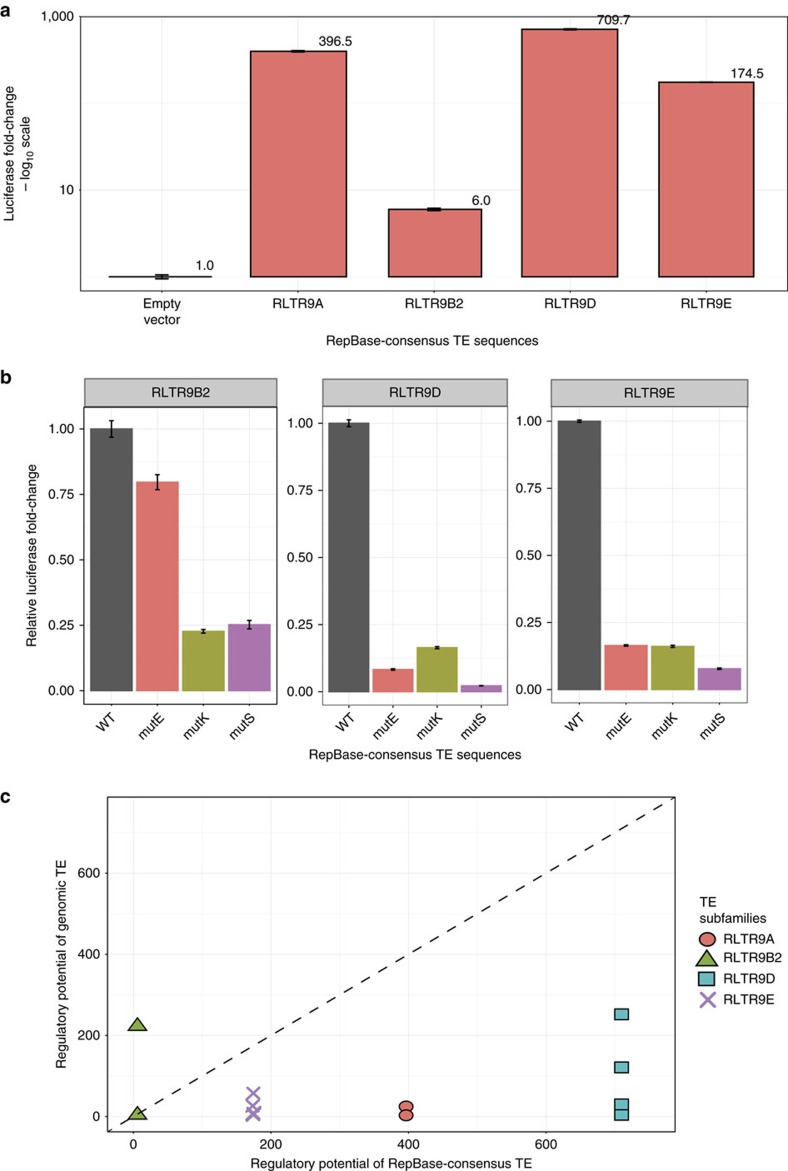
Regulatory potential of the RepBase-consensus TE sequence in mouse ESCs. (**a**) Luciferase fold change of the RepBase-consensus TE sequence of the four candidate RLTR9 TE subfamilies, in mouse ESCs. The luciferase fold change values represent the average of three technical replicates, and are plotted on a log_10_ scale. Error bars represent s.d. (**b**) Luciferase fold change of the RepBase-consensus TE sequence when individual motifs were mutated normalized to the unmutated TE sequence's regulatory potential. Error bars represent s.d. (**c**) Comparing the regulatory potential of genomic and RepBase-consensus TE sequences in mouse ESCs. Comparison of the regulatory potential of genomic (*y* axis) and RepBase-consensus (*x* axis) TE sequences. The diagonal represents *y*=*x* on this plot.

The ability of TEs to act as transcriptional enhancers is well documented^6^,^10^,^11^,^42^,^43^,^44^,^45^. However, it is still unclear whether TEs enter the genome with its regulatory potential and spread the sequence around the genome, or whether TEs acquire the regulatory potential after inserting into the genome^18^,^46^. As a proxy for the ancestral state of the TE, we used the RepBase-consensus sequence of each TE subfamily^14^,^29^,^31^,^47^. We synthesized the RepBase-consensus sequence of the four RLTR9 subfamilies and assayed their regulatory potential in mouse ESCs using luciferase assay (see Methods; Supplementary Table 9). Interestingly, the RepBase-consensus TE sequences also enhanced luciferase gene expression in mouse ESCs (Fig. 5a). Assuming the RepBase-consensus sequence to be a proxy for the ancestral state of the TE sequence, this result suggests that the ancestral sequence of the four RLTR9 subfamilies are likely to have entered the genome with the ability to regulate gene expression in ESCs.

To investigate the evolution of EKS module in these TE subfamilies, we scanned the RepBase-consensus sequence for TF-binding motifs (see Methods; Supplementary Table 10). Indeed, we observed the same EKS motif module in the RepBase-consensus sequence too (Supplementary Fig. 13). To test the cooperativity among the EKS motifs in the RepBase-consensus sequence, we mutated individual motifs in the RepBase-consensus sequence of RLTR9B2, RLTR9D and RLTR9E (see Methods; Supplementary Tables 10 and 11) and quantified their regulatory potential in mouse ESCs. We observed that mutating one motif in the EKS module results in a large decrease in the regulatory potential of the RepBase-consensus sequence (Fig. 5b). Here the only exception is mutating Esrrb in RLTR9B2. This result can be explained by the low-scoring Esrrb motif in this sequence (motif score of 1.97 in the RepBase-consensus sequence compared to 5.77 in the genomic TEs) that might reduce the synergistic interaction with the Klf4- and Sox2-binding sites in the EKS module. Taken together, assuming the RepBase consensus to be an approximation of the ancestral TE, our results indicate that RLTR9B2, RLTR9D and RLTR9E subfamilies might have entered the genome with modules of EKS-binding sites and the potential to regulate gene expression.

Next, we sought to understand the evolution of the *cis*-regulatory module and regulatory potential in TEs from the ancestral to present-day copies. We compared the regulatory potential (that is, luciferase fold change) between the genomic TE and RepBase-consensus sequences (Fig. 5c). Most of the genomic TEs had similar TF-binding motifs and shared on average 82.6% sequence identity (range: 51.42–90.26) with its corresponding RepBase-consensus sequence (Supplementary Fig. 14). Among the sequences in the four TE subfamilies that we characterized, the majority (10 out of 12) of the genomic TEs had lower regulatory potential compared to their RepBase-consensus sequence (Fig. 5c). If the genomic TEs had the same regulatory potential as the ancestral TE sequence, then a perfect correlation between the luciferase fold change values of genomic and ancestral TE sequences can be expected. However, we observe that most of the genomic copies have lower regulatory potential compared to their respective ancestral sequences. Interestingly, one RLTR9B2 genomic element had higher regulatory potential compared to its ancestral sequence, while the other RLTR9B2 element in our study had the same regulatory potential. We analysed the sequence of the RLTR9B2 genomic copy that had a higher regulatory potential, and observed that the Esrrb motif in the genomic copy with a higher score than the corresponding motif in the RepBase-consensus sequence. This suggests that the genomic copy might have evolved a stronger Esrrb motif that interacts synergistically with Klf4 and Sox2 to result in the high regulatory potential. In addition, we observed that the effect of mutations to the motifs is different in the RepBase-consensus sequence and the genomic TE sequence (Supplementary Fig. 15). On the one hand, RLTR9D shows perfect correlation in the comparison, while for RLTR9B2 and RLTR9E the effect of different motifs is different between the genomic and RepBase-consensus sequences.

## Discussion

*Cis-*regulatory modules are involved in specifying cell types^17^,^48^,^49^ as they contain binding sites for multiple TFs of a particular biological pathway. The presence of a module of TF-binding sites enables the coordinated effect of multiple TFs to be applied to a particular biological pathway or biochemical pathway. As seen in Britten and Davidson's model^15^,^16^, TEs form an efficient evolutionary mechanism for the coordinated evolution of *cis*-regulatory modules at multiple sites in the genome. At the outset, Barbara McClintock as well as Eric Davidson and Roy Britten demonstrated the role of TEs in gene regulation^1^,^2^,^15^,^16^. This model has since been robustly validated with numerous studies showing that TEs contain binding sites for various TFs in various cell types^6^,^8^,^9^,^10^,^12^,^18^,^50^. However, these studies have focused on TEs' contribution to individual TF-binding landscape.

TEs by virtue of their inherent regulatory nature and their ability to transpose make them an excellent candidate for rewiring gene regulatory networks. For the genome to evolve the same *cis-*regulatory module without TEs, it would require the coordinated and independent evolution of the same regulatory module sequence across multiple genomic locations. Sequence mutation is a common mechanism for evolving new sequences. However, the biggest limitation of this mode of regulatory sequence evolution in comparison with TEs is that sequence mutation occurs randomly, and therefore it is not a suitable mechanism to evolve the same regulatory sites across the genome coordinately. Alternatively, several insertions of a TE that contains a *cis*-regulatory module can quickly rewire the existing gene regulatory network by bringing new genes under the control of the regulators.

Although it is widely accepted that TEs can contribute TF-binding sites to the genome, it still remains to be understood how TEs evolve these TF-binding sites^18^,^51^,^52^. The binding sites in these TEs could arise in two ways. First, the ancestral TE entered the genome with the TF-binding sites and spread this sequence across the genome as it transposed. Alternatively, the ancestral TE lacked the TF-binding sites, but one of its insertions that were still capable of transposing evolved the TF-binding site and spread the sequence while transposing. In the context of a module of TF-binding sites, a TE that is capable of transposing is a much more efficient evolutionary mechanism for the rapid spread of modules of TF-binding sites.

In this study, we found certain TE subfamilies (that is, RLTR9B2, RLTR9D and RLTR9E) that were enriched for multiple TFs-binding sites (specifically, Esrrb-, Klf4- and Sox2-binding motifs), making them candidates to study TEs' role in evolving *cis-*regulatory modules in the genome. We demonstrate that genomic TEs from these three subfamilies are capable of enhancing gene expression, and more importantly function as a module to regulate gene expression (Fig. 4b). Using the RepBase-consensus sequence^29^ as an approximation of the ancestral TE sequence^30^,^31^, we not only found the same EKS module in the RepBase-consensus sequences (Supplementary Fig. 13), but also demonstrate that these binding sites function as modules (Fig. 5b), just like the genomic copies of these TE subfamilies. Together, these results suggest that the genomic copies of RLTR9B2, RLTR9D and RLTR9E that contain EKS modules most likely have inherited the EKS module and the ability to regulate gene expression from their ancestral state. Since we cannot analyse the ancestral state of these TEs, the RepBase-consensus sequence is an approximation for the TE's ancestral state. Therefore, our results are indicative of the ancestral state of these TEs also being capable of regulating gene expression.

Genomic TEs most likely inherited the regulatory potential and the module from the ancestral TEs. However, we also observe that the genomic TEs have lower regulatory potential in comparison with the ancestral state. This is consistent with the hypothesis that when TEs are co-opted to serve a host function their potential can be tinkered (under neutral evolution—Supplementary Fig. 16) to fit the host's requirements). From our transcriptome analyses, we identified that these TEs could be regulating certain genes in a mouse ESC-specific manner (Fig. 3c). The TEs we characterize in this manuscript are candidates for regulating gene expression in a mouse ESC-specific manner, thereby distinguishing ESCs in mice from ESCs in other species. *Akap12* is an example of a gene regulated by RLTR9E specifically in mouse ESCs. Mouse and human ESCs are known to have differences in their signalling pathways^39^; *Akap12* is one gene involved in signalling pathways and specifically upregulated in mouse ESCs. Taken together, our results highlight a role for TEs in depositing modules of pluripotency TF-binding sites and contribute towards mouse ESC-specific gene expression patterns.

## Methods

### Processing data for pluripotency TF binding in mouse ESCs

We used ChIP-seq peaks from Chen *et al*.^19^ for five pluripotency TFs (Esrrb, Klf4, Nanog, Oct4 and Sox2; GEO accession number: GSE11431). The peaks were defined on mm8 genome assembly, so we extended the peaks to 200 bp and then mapped to mm9 genome assembly using the *liftOver* tool^53^ (requiring the *–minMatch* parameter set to 0.95). The total number of peaks used in the analyses for this paper is tabulated in Supplementary Table 1.

To identify TF-binding motifs, we used position-weight matrices for the five TFs from JASPAR^54^ and TRANSFAC^55^, and *patser*^56^ to scan for motifs. We selected all motifs (*P* value<0.001) that matched the motif's consensus.

### Defining clusters of TF-binding sites

To determine clusters of TF-binding sites (that is, ChIP-seq binding peaks or computationally predicted TF-binding motifs), we identified regions that had multiple TF-binding sites that were within 100 bp of each other. For ChIP-seq peaks, we used the centres of the peaks as the reference point for each peak, while for TF-binding motifs we used the starting position of the motif.

We overlapped these clusters of TF-binding sites with RepeatMasker-annotated TEs^57^ in mm9, to identify TEs that encode clusters of TF-binding sites using the *intersectBed* tool^58^. Next, we measured the enrichment of ‘*i*' TF-binding sites (which can be ChIP-seq peaks or computationally predicted TF-binding motifs) in TE subfamily ‘*j*', using a log odds ratio:


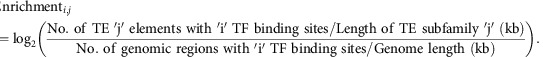


We plotted TE subfamilies that had at least 10 elements that overlapped genomic regions with ‘i' TF-binding sites.

### Estimating the evolutionary age of TE subfamilies

For the evolutionary analyses of these TE subfamilies, we used three methods. First, we used blast^28^ to align the RepBase-consensus sequence^29^ of each TE subfamily to other genome assemblies in the vertebrate lineage. We downloaded the genome assemblies from the UCSC Genome Browser^53^, and used the *blast* tool^28^. We identified no hits for the six candidate TE subfamilies in any of the genome assemblies at a threshold of 50% sequence identity and 1E–10 E-value.

Second, we used sequence divergence to estimate the substitution rate of each element in a particular TE subfamily using the Juke-Cantor evolutionary model^32^. To do this, we obtained the divergence (*p*) of each element compared to the RepBase-consensus sequence, from the RepeatMasker output (rmsk file) on the UCSC genome browser. The substitution rate was calculated using the formula:


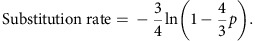


Finally, we used intact ERV sequences of the six TE subfamilies interest in the genome to estimate when the candidate TE subfamilies entered the genome. This approach is based on the hypothesis that LTRs for a particular ERV sequence are identical at the time of insertion and then undergo independent sequence evolution. We identified 107 pairs of intact TEs for the six subfamilies that were within 7 kb apart and then estimated the sequence divergence between the two LTR sequences using *blast2* (ref. 59). We used the lower and upper bounds for the mutation rate, between mouse and rat as 1.2 and 0.05% per site, per million years (Supplementary Information from refs 5, 33).

### Epigenomic analyses of TEs bound by multiple TFs

We analysed various histone modification marks and DNA methylation in mouse ES (ES-E14) cells to determine the epigenetic signature of TEs (from the six candidate TE subfamilies) bound by various number of TFs (that is, 0, 1 and ≥2). We downloaded ChIP-seq data for histone modification marks (H3K4me1, H3K4me3, H3K27ac, H3K9me3 and H3K36me3) from the mouseENCODE project^60^ and used P300 ChIP-seq data from Creyghton *et al*.^61^ All the reads were aligned to the mm9 genome, using *bwa*^62^. For DNA methylation, we generated complete DNA methylomes of mouse ESCs (E14)^10^,^60^,^63^ at single CpG resolution^64^. To obtain the epigenetic signature, we categorized TEs based on the number of TFs it had bound on it (that is, 0, 1 and 2 or more) and averaged the normalized read density for each epigenetic mark in 50 bp bins across a 3 kb region (1.5 kb flanking on either side of the center of the TE). For cell-type specificity, we analysed the epigenetic data in mouse ES (E14) cells, lymphoblastoid (Ch12) and erythroleukemia (Mel) cells from the mouseENCODE project^10^,^60^,^63^ in the same manner as described earlier.

### Identifying mouse ESC specifically expressed genes

We used transcriptome data for human and mouse ES, lymphoblastoid and erythroleukemia cells from the mouseENCODE project^60^ for human and mouse 1:1 orthologous genes. To identify mouse ESC specifically expressed genes, we used DESeq^65^, which is a software application that identifies differentially expressed genes based on the read-count assigned to a particular gene. We performed pair-wise comparisons between all the cell types and the mouse ES cell, to identify genes that were differentially expressed between mouse ESCs and the other cell types. On average, we found that 6,384 genes were differentially expressed (adjusted *P* value<0.1) compared to mouse ESCs. Among these, 1,868 genes were upregulated in specifically in mouse ESCs (Supplementary Fig. 4). We then used the *closestBed* tool^58^ to associate each TE with a mouse ESC specifically expressed gene.

### CRISPR-mediated deletion of RLTR9E

From the transcriptome analyses that we performed, we identified that *Akap12* was specifically expressed in mouse ESCs (Fig. 3c). *Akap12* also contains in its first intron, a RLTR9E element that is bound by multiple TFs *in vivo* in mouse ESCs. We sought to delete this intronic TE to determine its effect on the *Akap12*'s expression level. Two sets of guide RNAs were designed to specifically target and delete RLTR9E. After testing the specificity of the guide RNAs on genomic DNA, the RW4 cells were targeted with the guide RNAs to delete the RLTR9E element. Clones were screened to identify cells with homozygous deletions for the RLTR9E element, and subsequently confirmed by Sanger sequencing. This project was done at the Genome Engineering iPSC Core at Washington University School of Medicine.

To determine the effect of the RLTR9E-derived TF-binding module on the expression of *Akap12*, RNA was extracted from clones with the CRISPR deletion, and wild-type cells—two biological replicates each—and converted to cDNA using the iScript Reverse Transcription kit (Bio-Rad). We quantified the expression level of *Akap12* transcript in wild-type mouse ESCs (RW4 cell line) and cells with the CRISPR-mediated deletion, using the SYBR Green Master Mix (Bio-Rad). The quantification of the expression levels of *Akap12* gene, and other neighbouring genes as control (*Lrp11* and *Nup43*), was performed in the wild-type and CRISPR-deleted cells (two biological and three technical replicates each). We used *Gapdh* as the normalizing gene for these experiments (primers listed in Supplementary Table 12). Normalized expression levels (2^ΔΔCt^) for each gene were calculated by comparing the cycle-time (Ct) values of each gene to *Gapdh* Ct levels (ΔCt) in the same replicate, and then comparing ΔCt values between wild-type and CRISPR^−/−^ cells (ΔΔCt). Results are representative of three independent experiments.

### Luciferase assay in mouse ESCs (RW4)

For experimental validation of the enhancer function of TEs in mouse ESCs, we used the RW4 cell line, and cultured it as previously described^66^. RW4 cells were cultured in 0.1% gelatin-coated Petri dishes in culture medium containing DMEM (Invitrogen) supplemented with 10% fetal bovine serum (Gibco), 10% neonatal calf serum (Hyclone), nucleosides (Sigma), 1,000 U ml^−1^ leukemia-inhibiting factor (Sigma) and 0.1 mM β-mercaptoethanol (Gibco).

To estimate the regulatory potential of TE and non-TE sequences in mouse ESCs, we used the Dual-Glo luciferase assay (Promega). For the luciferase assay, we cloned TEs into the pGl4.23 vector (containing firefly luciferase; Supplementary Tables 5A–C and 9). We co-transfected the TE-plasmid with pRL-TK (containing renilla luciferase) using lipofectamine (X-GENE-HD at 1 μl for 1 μg of plasmid DNA) into RW4 cells plated in 0.1% gelatin-coated 96-well plate. After 24 h, we assayed the luciferase levels according to the Dual-Glo reporter assay system (Promega).

We performed each luciferase experiment in triplicate, and repeated each experiment three times. The luciferase fold change is estimated as the ratio of firefly and renilla luciferase for each TE, and then normalized by the empty vector (pGl4.23 with no insert).

### CRE-seq—a massively parallel reporter assay

CRE-seq is a massively parallel reporter assay^41^. This technique uses a high-throughput cloning technique to clone a pool of synthesized oligomers into plasmid vectors containing the reporter gene (which in this case was *dsRed *and a *Pou5f1* minimal promoter.). Each test TE CRE sequence (19 genomic TEs listed in Supplementary Table 7; each was associated with single and triple mutants based on Supplementary Table 8) was 82 bp long and was associated with eight unique barcode sequences (CRE barcode), which is equivalent to eight technical replicates. The cloned plasmid vectors were transfected into the RW4 cell line (with three replicates) using lipofectamine (as described above). The RNA was collected using the Ambion PureLike kit and treated with DNase. The RNA was then converted to cDNA for subsequent library preparation and sequencing. In addition, we sequenced the DNA pool that was used for transfection, for normalizing the RNA counts.

We sequenced each CRE barcode to a depth of 3,000 × coverage, and retrieved on average 96% of the CRE barcodes. The CRE-seq expression levels were correlated with the luciferase assay (*R*^2^=0.43); the low correlation score can be attributed to the technical differences in the two assays, including differences in the lengths of the sequences tested (82 bp in CRE-seq compared with ∼300 bp in the luciferase assay). We obtained high correlation between the three biological replicates (*R*^2^=0.9). The expression level of a CRE sequence is measured as the average of the ratio of sequencing read counts of cDNA to DNA, per CRE barcode, across biological and technical replicates. We normalized the expression level of each CRE to basal CREs (that is, empty vector) to make comparisons between CREs. To determine the impact of mutations to the EKS module on the wild-type CRE's expression, we normalized the expression level of the mutant sequence to its corresponding wild-type sequence's regulatory potential.

### Site-directed mutagenesis

We used the QuikChange site-directed mutagenesis protocol (Agilent Technologies) to mutate individual TF-binding motifs in the RepBase-consensus sequence. We designed primers to target each motif on plasmid constructs and incorporate upto four mutations per motif (Supplementary Tables 6B and 11).

Mutations were introduced at positions in the motif that had the highest information content and were replaced by the least informative nucleotide at that position. We verified the primers to ensure that the mutations did not create a new TF-binding motif, using the TOMTOM tool from the MEME suite of motif analysis^67^.

### Synthesizing RepBase-consensus sequences

We used gBlocks Gene Fragments from IDT to synthesize the RepBase-consensus sequences listed in Supplementary Table 9.

### Data availability

The data sets generated during and/or analysed during the current study are available from the corresponding author on reasonable request.

## Additional information

**How to cite this article:** Sundaram, V. *et al*. Functional *cis*-regulatory modules encoded by mouse-specific endogenous retrovirus. *Nat. Commun.* **8**, 14550 doi: 10.1038/ncomms14550 (2017).

**Publisher's note:** Springer Nature remains neutral with regard to jurisdictional claims in published maps and institutional affiliations.

## Supplementary Material

Supplementary Information Supplementary Figures 1-16 and Supplementary Tables 1-12
