# Supplementary material for: Functional cis-regulatory modules encoded by mouse-specific endogenous retrovirus
Source: Nat Commun. 2017 Mar 28;8:14550. doi: 10.1038/ncomms14550 (PMC5379053; doi:10.1038/ncomms14550)
Supplement: Supplementary Information — Supplementary Figures 1-16 and Supplementary Tables 1-12 [file ncomms14550-s1.pdf]

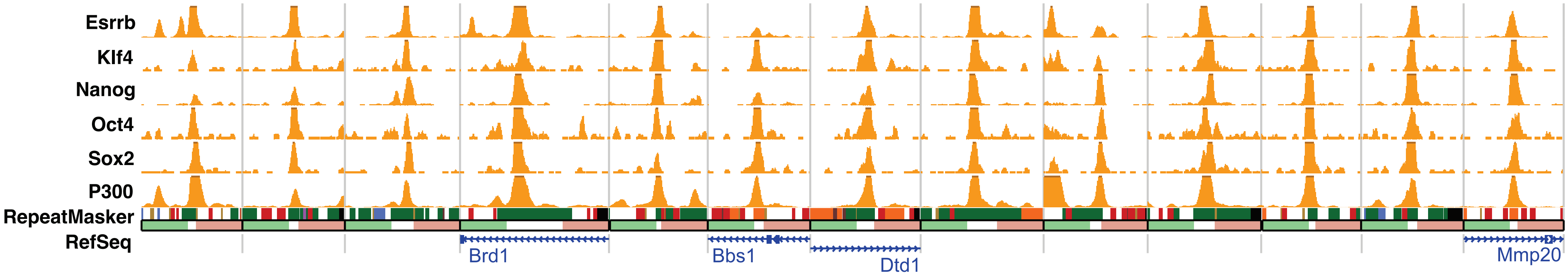

Supplementary Figure 1: Gene-set view (WashU Epigenome Browser - http://epigenomegateway.wustl.edu/browser) of 13 TEs that are bound by two or more TFs (yellow tracks: normalized read density of ChIP-seq). The view is centered on the TE, and shows 2.5 kb upstream (green bar) and downstream (red bar).

A

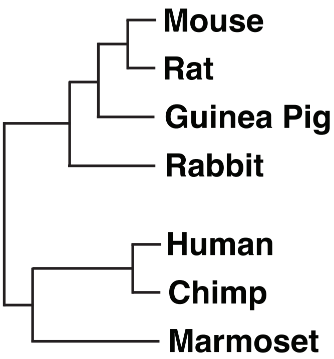

B

	RLTR9A	RLTR9B2	RLTR9D	RLTR9E	RLTR13D1	RLTR13D6
Mouse	✓	✓	✓	✓	✓	✓
Rat	✗	✗	✗	✗	✗	✗
Guinea Pig	✗	✗	✗	✗	✗	✗
Rabbit	✗	✗	✗	✗	✗	✗
Human	✗	✗	✗	✗	✗	✗
Chimp	✗	✗	✗	✗	✗	✗
Marmoset	✗	✗	✗	✗	✗	✗

Supplementary Figure 2: (A) Identifying in which species the six TE subfamilies that contain multiple TFs' binding sites exist. To do this, we used BLAST (Altschul SF, J Mol Biol, 1990) to search for the presence of each TE's RepBase-consensus sequence (Jurka J, Curr Opin Struct Biol, 1998) in the genomes of various species in the vertebrate phylogenetic tree. (B) We tabulated the results of the BLAST-search for the six TE subfamilies (columns) in the seven vertebrate species (rows). Tick-marks represent the present of the sequence in the genome, while cross-marks represent the absence of the sequence in the genome. Surprisingly, we only found the sequences in the mouse genome, and not in any other genome, including the closest relative, rat.

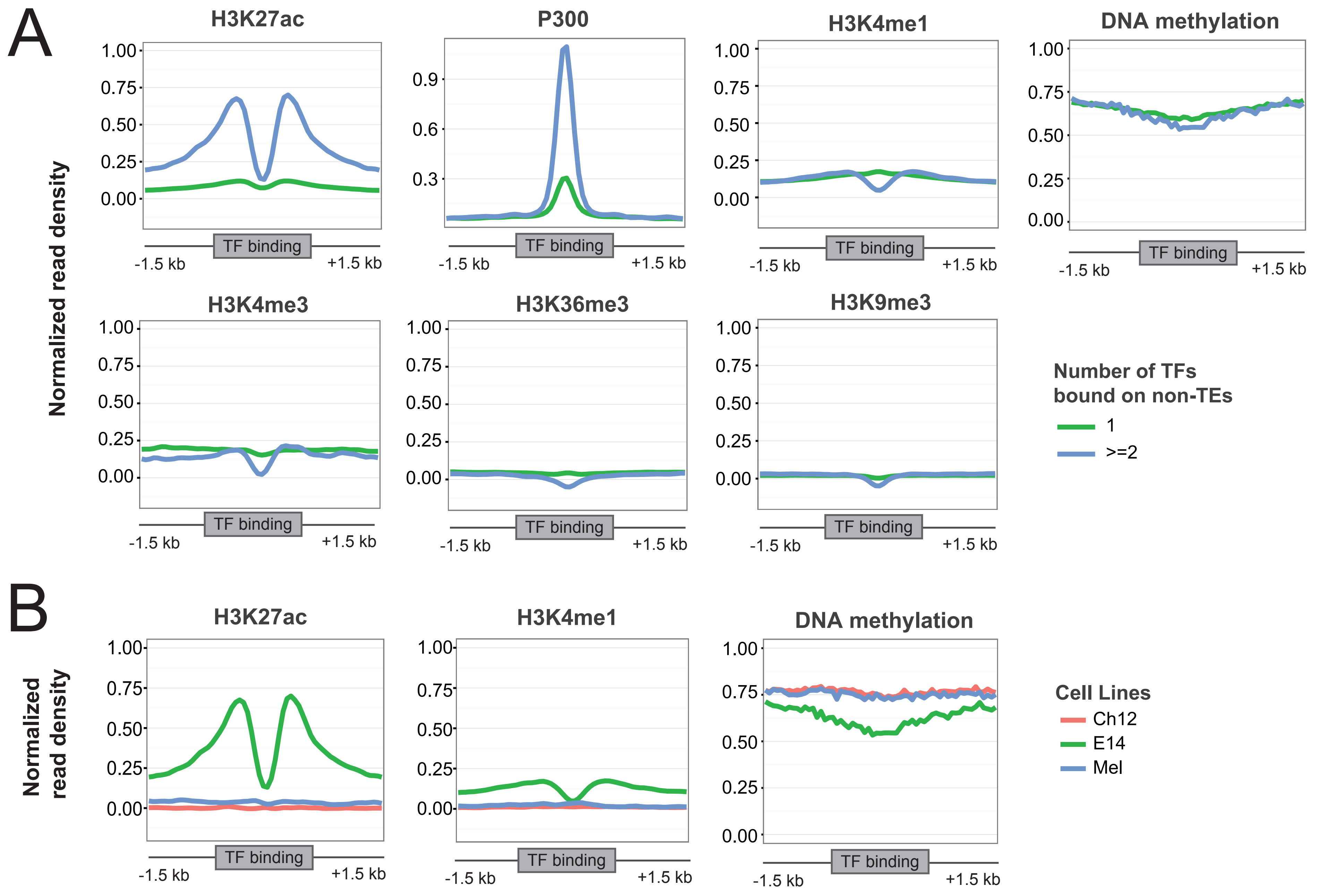

Supplementary Figure 3: Epigenetic signature of non-TE genomic regions that are bound *in vivo* by 1 TF, or a cluster of TFs (i.e, >=2 TFs). (A) Normalized read density on non-TE genomic regions for various epigenetic marks (panels). Each region was extended its center by 1.5kb upstream, and downstream. The regions are categorized by the number of TFs bound to the region - one TF or two or more TFs. (B) Comparing the epigenetic signature of non-TE genomic regions with two or more TFs bound in different mouse cell types - embryonic stem (E14), lymphoblastoid (Ch12), and erythroleukemia (Mel) cells.

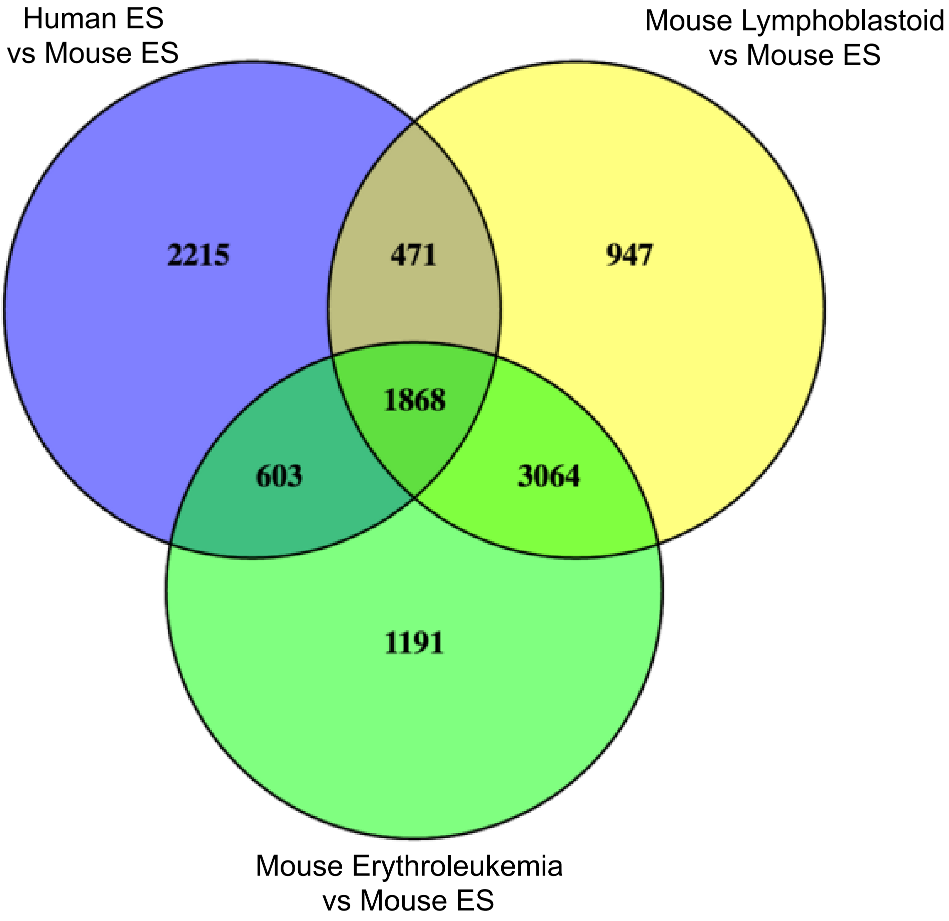

Supplementary Figure 4: Differentially expressed genes identified by DEseq (Anders S, et al., Genome Biology 2010), between three pairwise comparisons – human ES, mouse lymphoblastoid and mouse erythroleukemia versus mouse ES cells. Genes that were significantly upregulated in mouse ES cells (adjusted p-value < 0.1 in each pairwise comparison), and common between the three pairwise comparisons were used for further analyses (i.e., 1,868 genes). This figure was generated using *Venny* (Oliveros, JC, 2007-2015 – http://bioinfogp.cnb.csic.es/tools/venny/index.html).

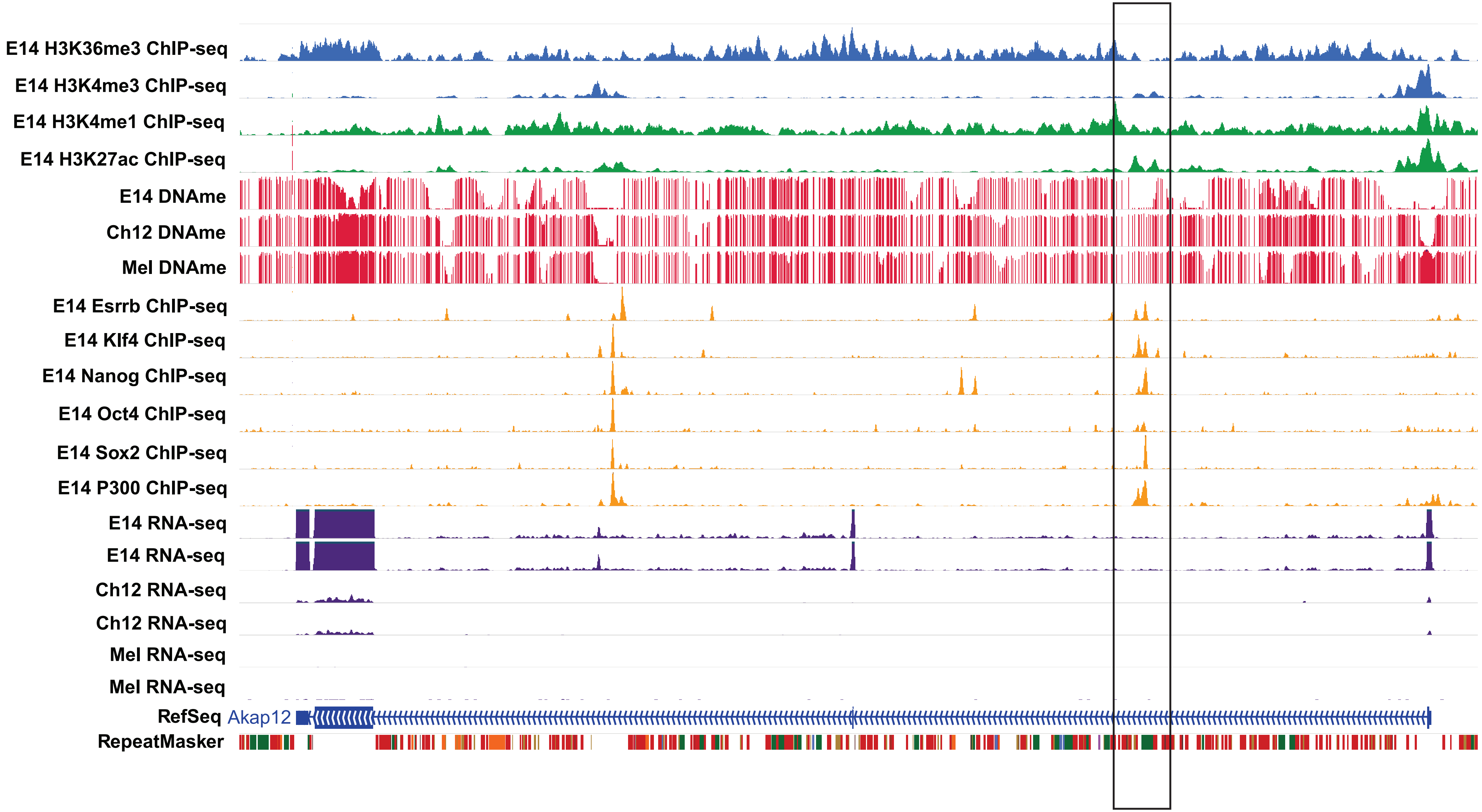

Supplementary Figure 5: Genome Browser (WashU Epigenome Browser) view of Akap12 gene that shows ES-specific expression in mouse ES cells (blue tracks: normalized read density for RNA-seq). Akap12 contains a RLTR9E element in its first intron, which is bound by four TFs (yellow tracks: normalized ChIP-seq read density) and is specifically demethylated (red tracks: single-CpG resolution of DNA methylation data) in mouse ES cells. Interestingly, in the second intron of Akap12 is a non-TE region that is also bound by the five TFs but is demethylated in lacks ES-specific hypomethylation. Additional ChIP-seq tracks for H3K36me3, H3K4me3, H3K4me1, and H3K27ac are listed on top.

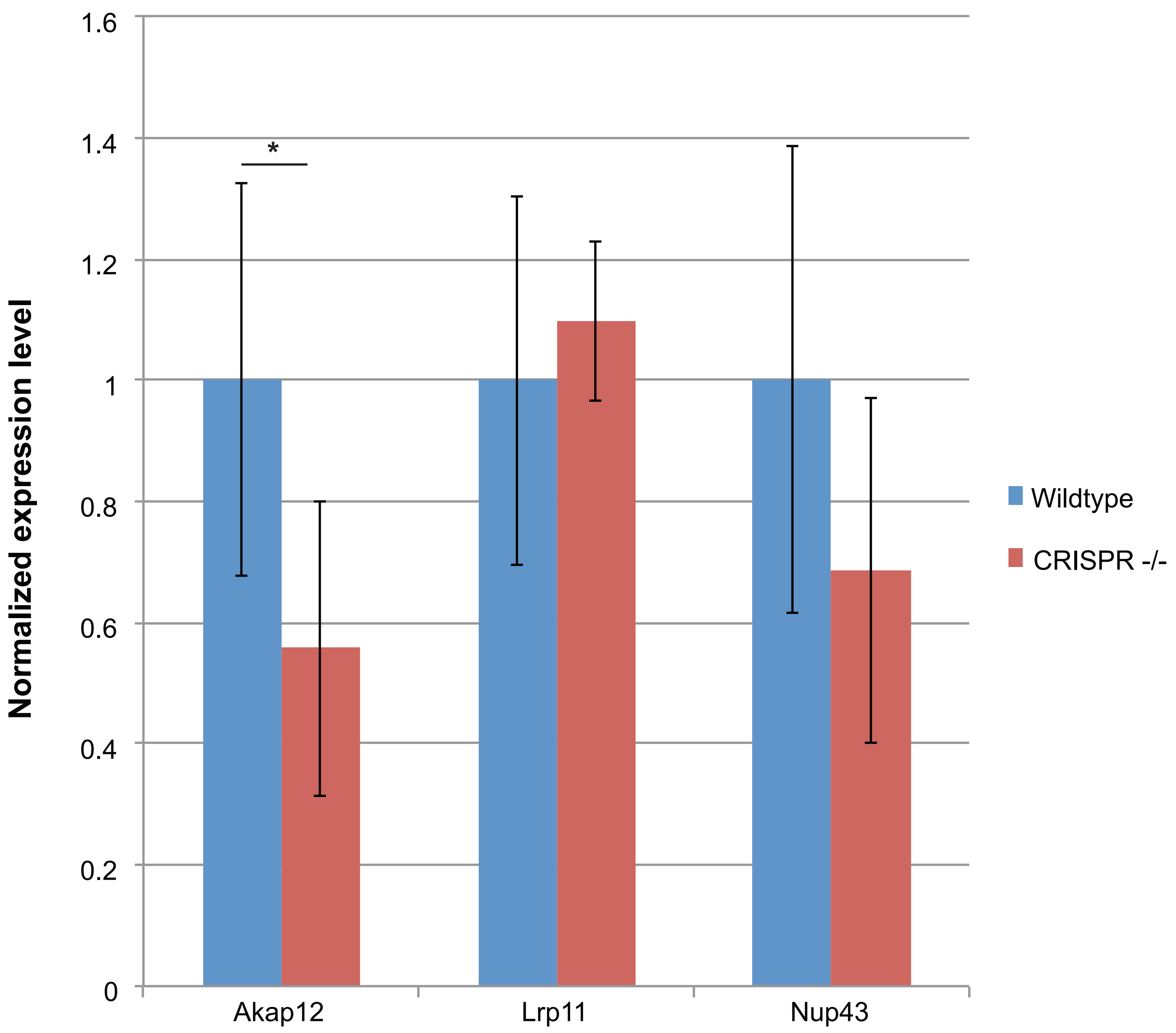

Supplementary Figure 6: Normalized expression level of Akap12 and two nearby genes - Lrp11 and Nup43. We performed qRT-PCR of these three genes in wildtype and CRISPR deletion clones (for RLTR9E deletion - labeled “CRISPR -/-”) in two biological and three technical replicates. The errorbars represent the standard deviation of the expression levels. Akap12 shows a ~45% reduction in expression level between the WT and CRISPR -/- clones, and is statistically significant as measured by a Student’s t-test (p-value < 0.05, denoted by *). Lrp11 and Nup43 does not show any statistically significant change in expression level between the Wildtype and CRISPR -/- clones.

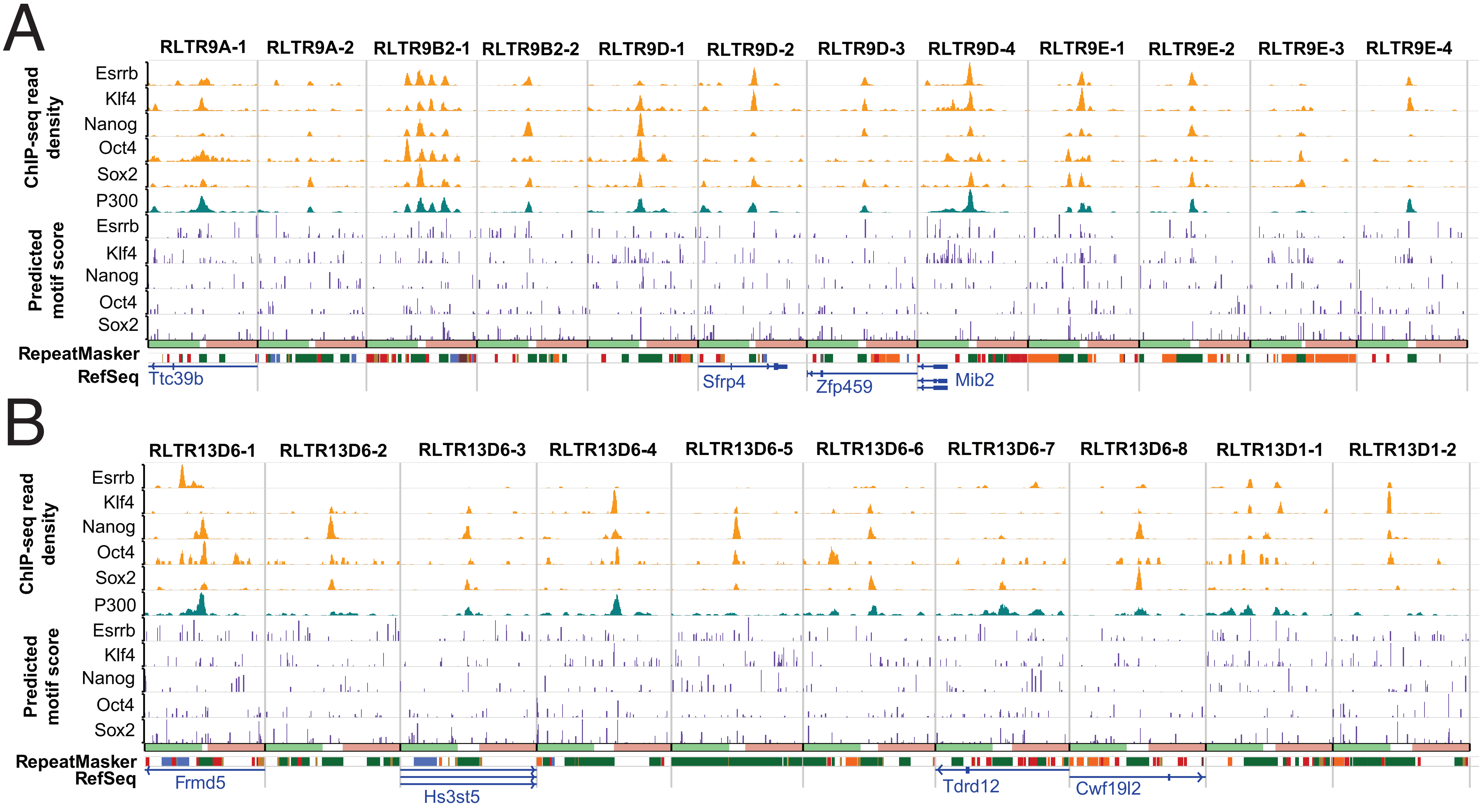

Supplementary Figure 7: Gene-set view (WashU Epigenome Browser) of the 22 TEs that were tested in a luciferase assay in mouse ES cells. The view is centered on the TE (horizontal white bar), and shows 2.5 kb upstream (green bar) and downstream (red bar). We show the ChIP-seq read density for the five TFs (yellow tracks), P300 (teal track), and predicted motifs (purple track) for (A) twelve elements from the RLTR9 subfamilies (i.e., RLTR9A, RLTR9B2, RLTR9D, and RLTR9E) and (B) ten elements from the RLTR13 subfamilies. The identifiers at the top of each view corresponds to the labels in Supplementary Table 5A.

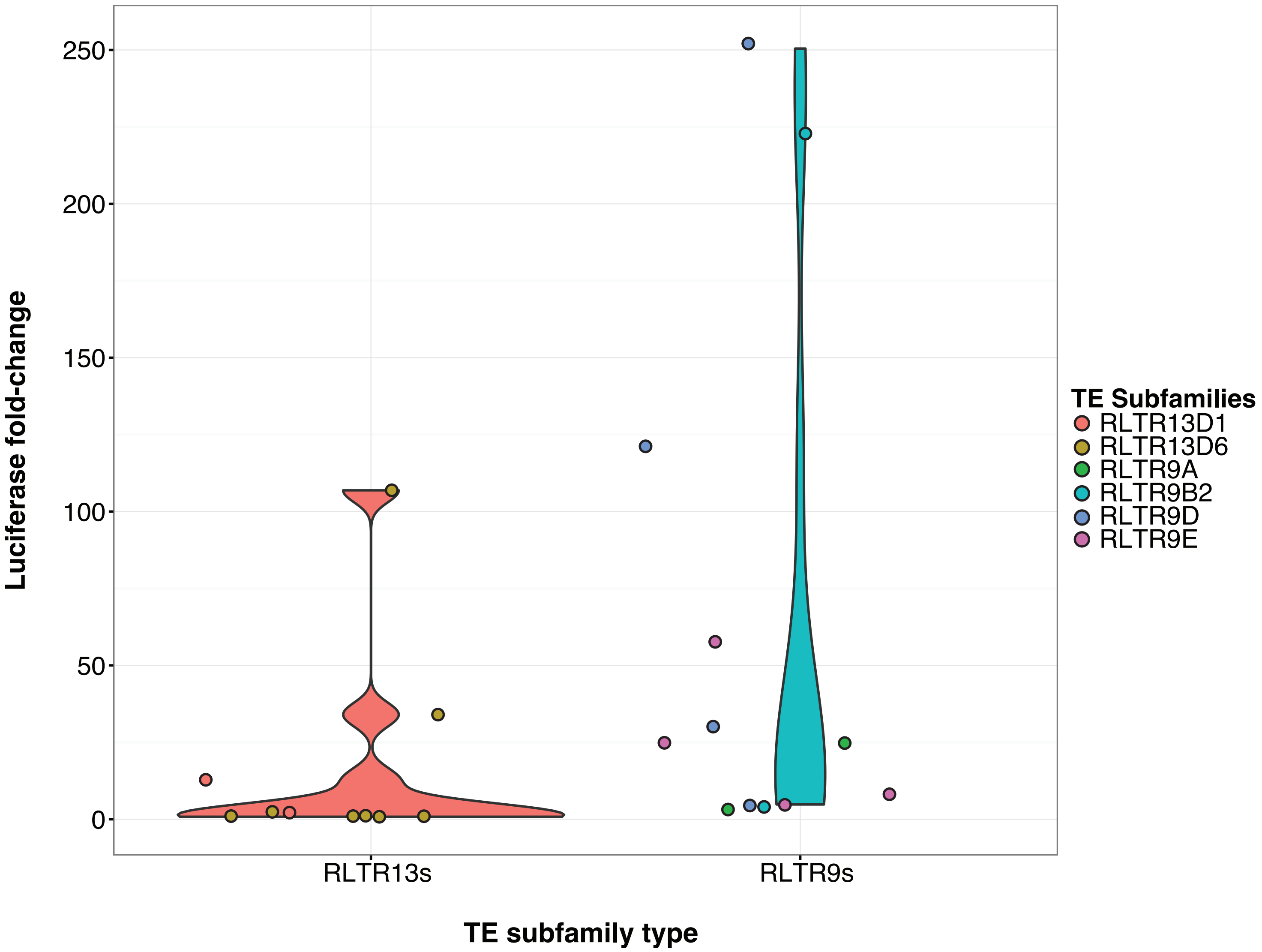

Supplementary Figure 8: Distribution of luciferase-fold change values for 22 different TEs (each dot) that we experimentally tested in the luciferase assay in mouse ES cells. On the x-axis are the two categories of TE subfamilies (i.e, RLTR13 vs RLTR9). Overall, we observe that the RLTR13 subfamilies (RLTR13D1 and RLTR13D6) have lower luciferase fold-change values, than the RLTR9 subfamilies (RLTR9A, RLTR9B2, RLTR9D, and RLTR9E).

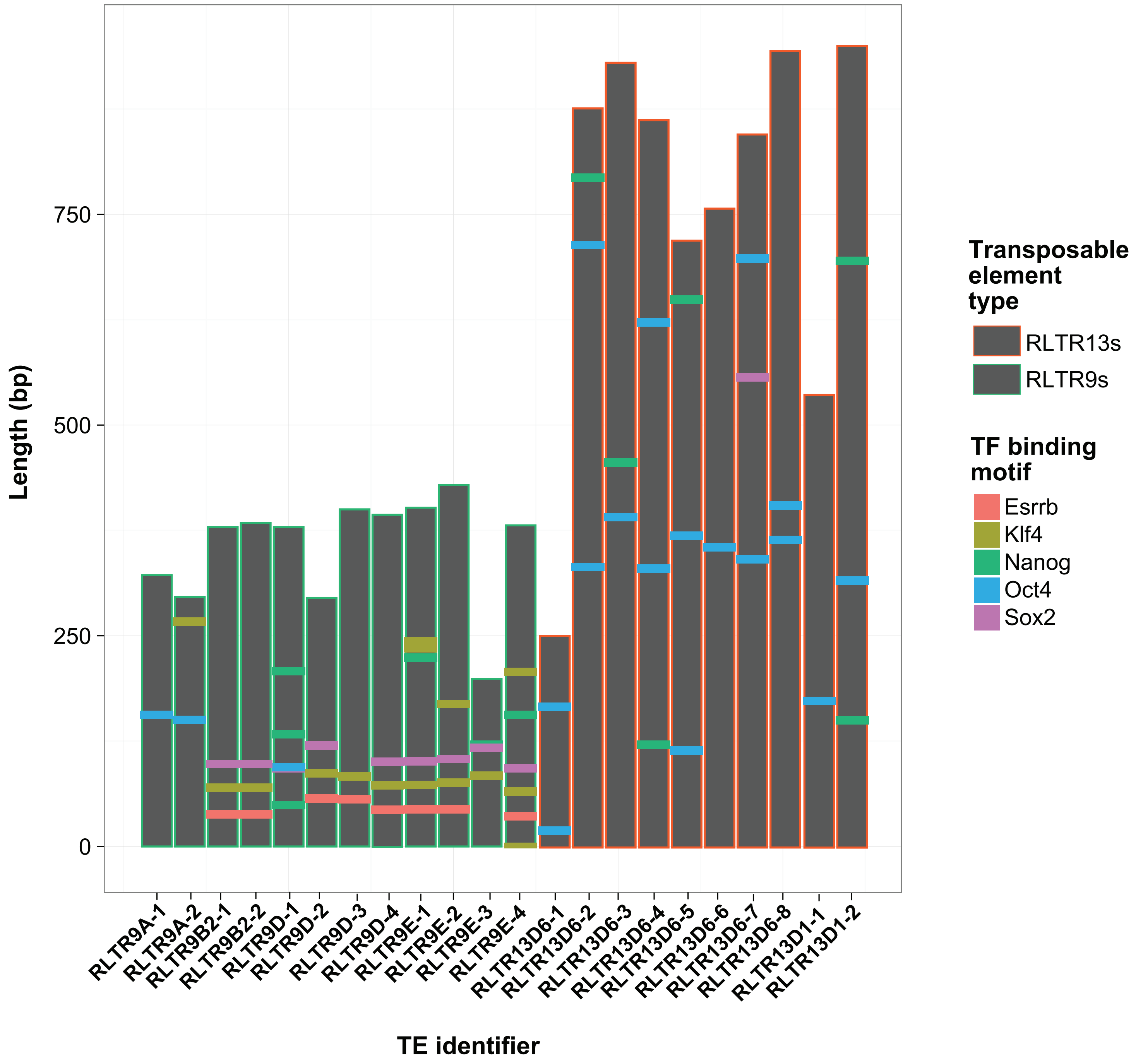

Supplementary Figure 9: Motif-annotations in TEs that we tested in the luciferase assay (Figure 4A). The TEs (x-axis) belong to two types of TE subfamilies - RLTR9s (green outline) and RLTR13s (orange outline). Each TE is represented by a bar, whose height represents the length (bp) of the TE (y-axis). The small bars in each TE represents an identified motif for the various pluripotency TFs (see legend).

A

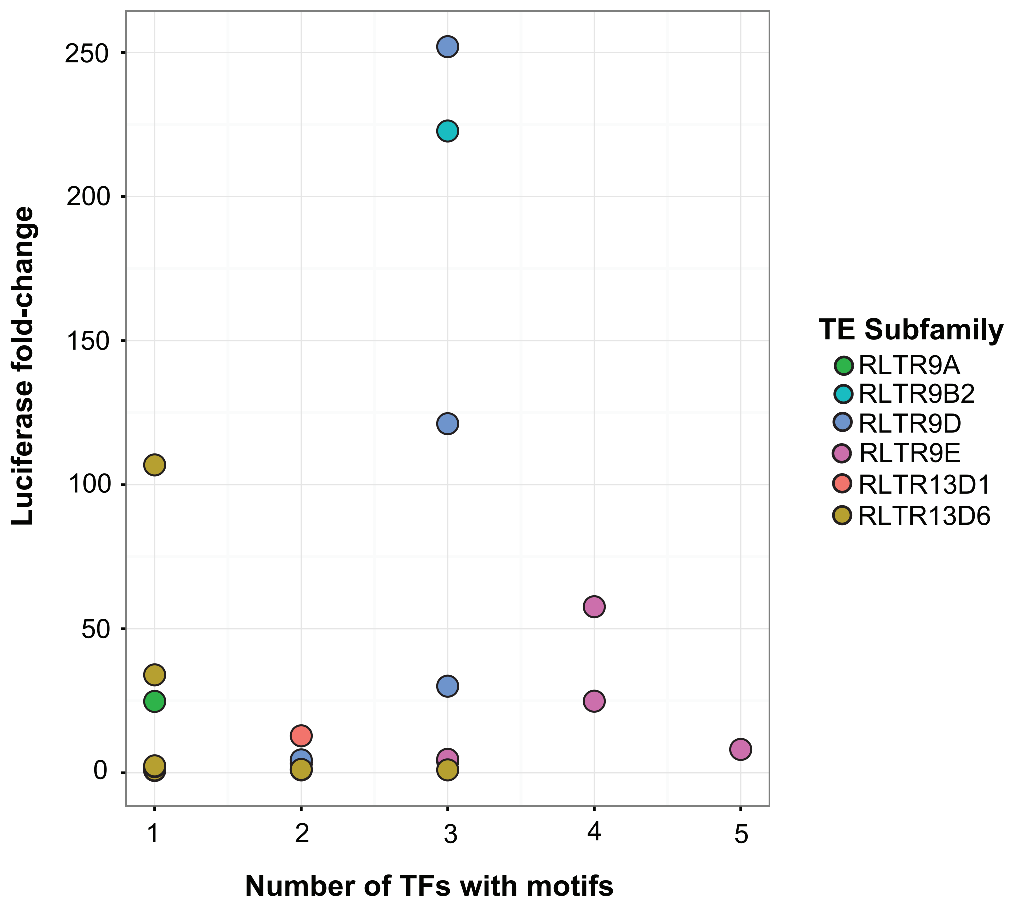

B

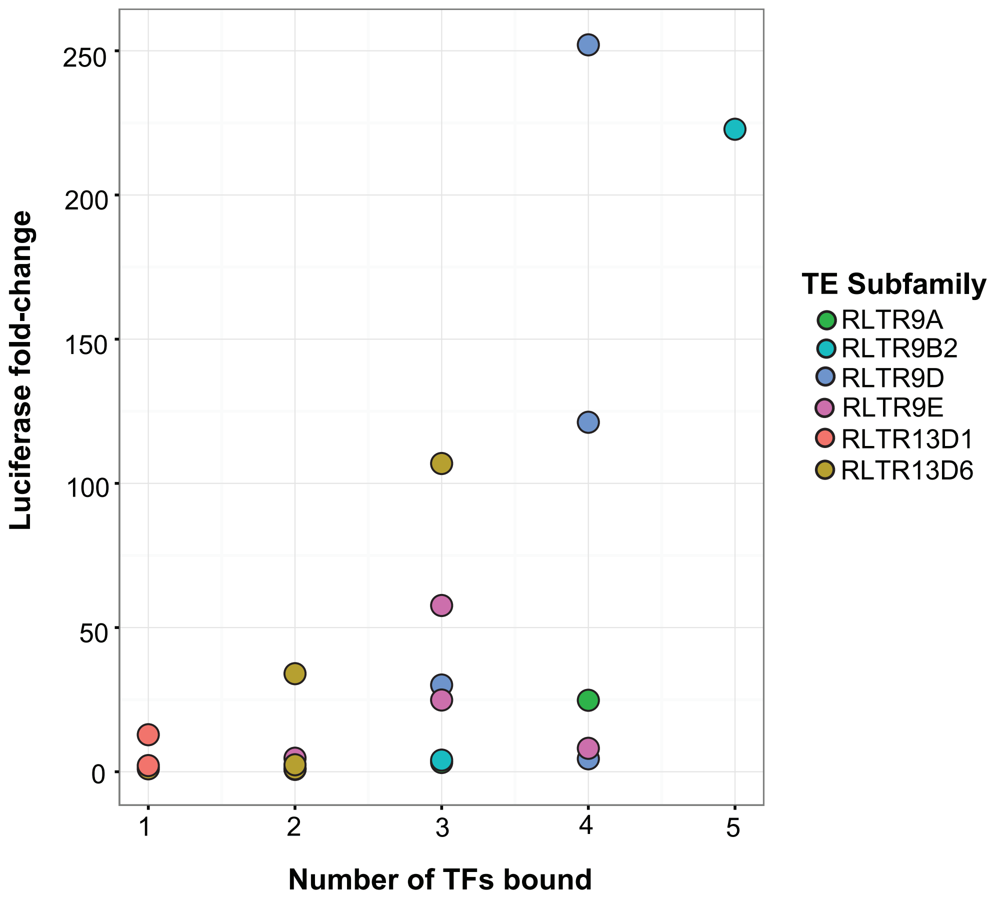

Supplementary Figure 10: Distribution of the regulatory potential (luciferase fold-change, y-axis) in mouse ES cells versus (A) the number of TFs with motifs predicted in the TE, and (B) the number of TFs bound to the TE. Here, we see that increasing the number of TFs with binding sites (motifs and peaks) corresponds with an increase in the luciferase expression driven by the TE. However, exceeding a certain limit corresponds with a decrease in the regulatory potential of the TE.

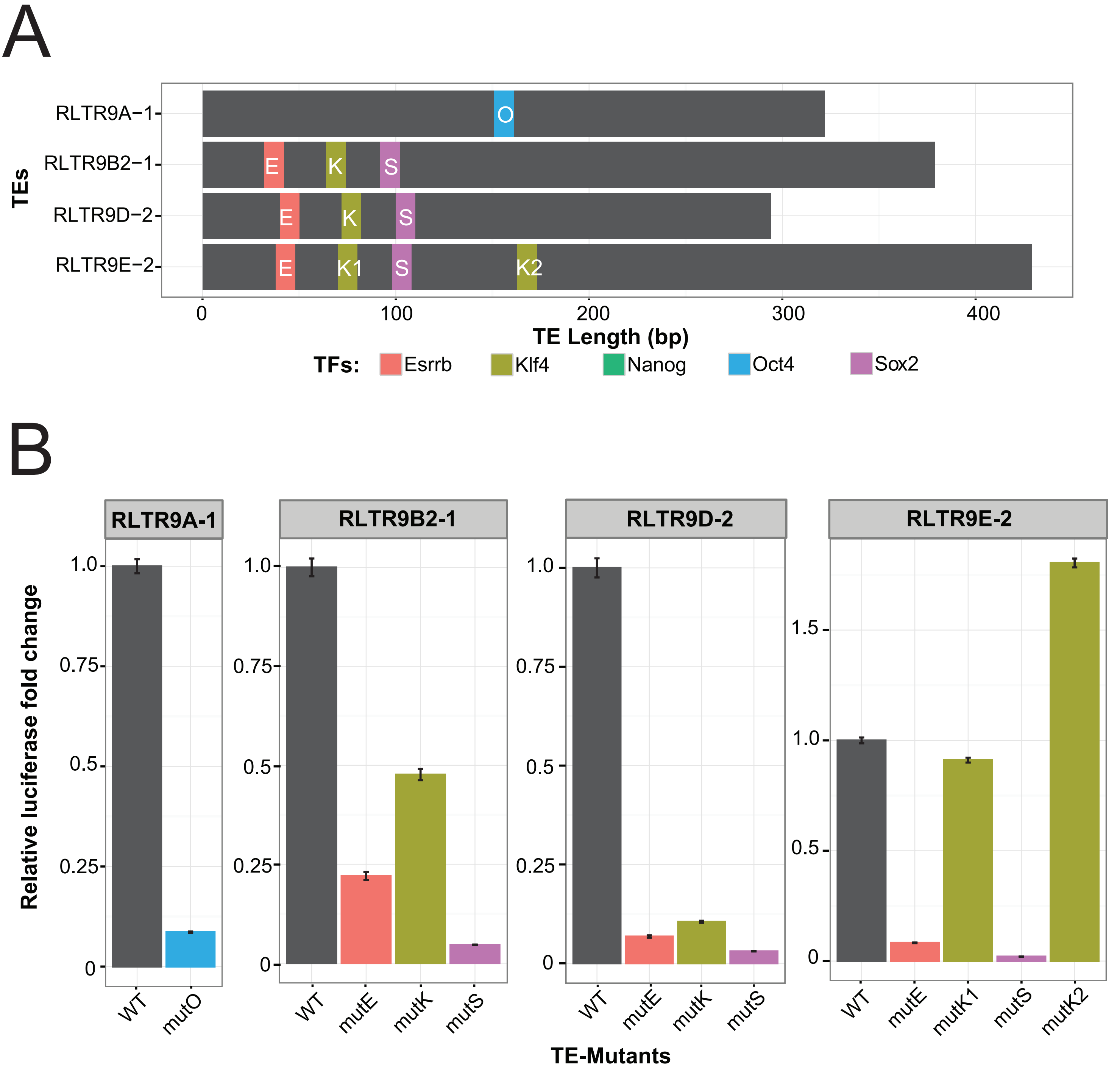

Supplementary Figure 11: (A) Motif-annotations in TE selected for site-directed mutagenesis and luciferase assay. The row names refer to the identifier of the TE (listed in Supplementary Table 5A). Each bar represents a TE, and is annotated with the motif predictions for the five pluripotency TFs. (B) Relative luciferase fold-change of each of the four TEs shown in (A) in which each motif was mutated. The luciferase fold-change of the mutant sequence was normalized to the wildtype (WT) luciferase fold-change for each TE, to show the difference in the regulatory potential. Error bars represent s.d. Overall, the reduction in regulatory potential caused by mutations to Esrrb (E), Klf4 (K) and Sox2 (S) indicates a synergistic relationship between the motifs. The contribution of all three motifs together is more than the sum of each motif's contribution. Additionally, we observe differences in the effect of the two Klf4 motifs (K1 and K2) to the regulatory potential of RLTR9E-2. It appears that K2 in RLTR9E-2 might have a repressive effect (Evans, P, et al., JBC 2007; Rowland, BD, et al., Nat. Cell Biol. 2005) on K1, since mutating K2 results in an increase in the regulatory potential of RLTR9E-2.

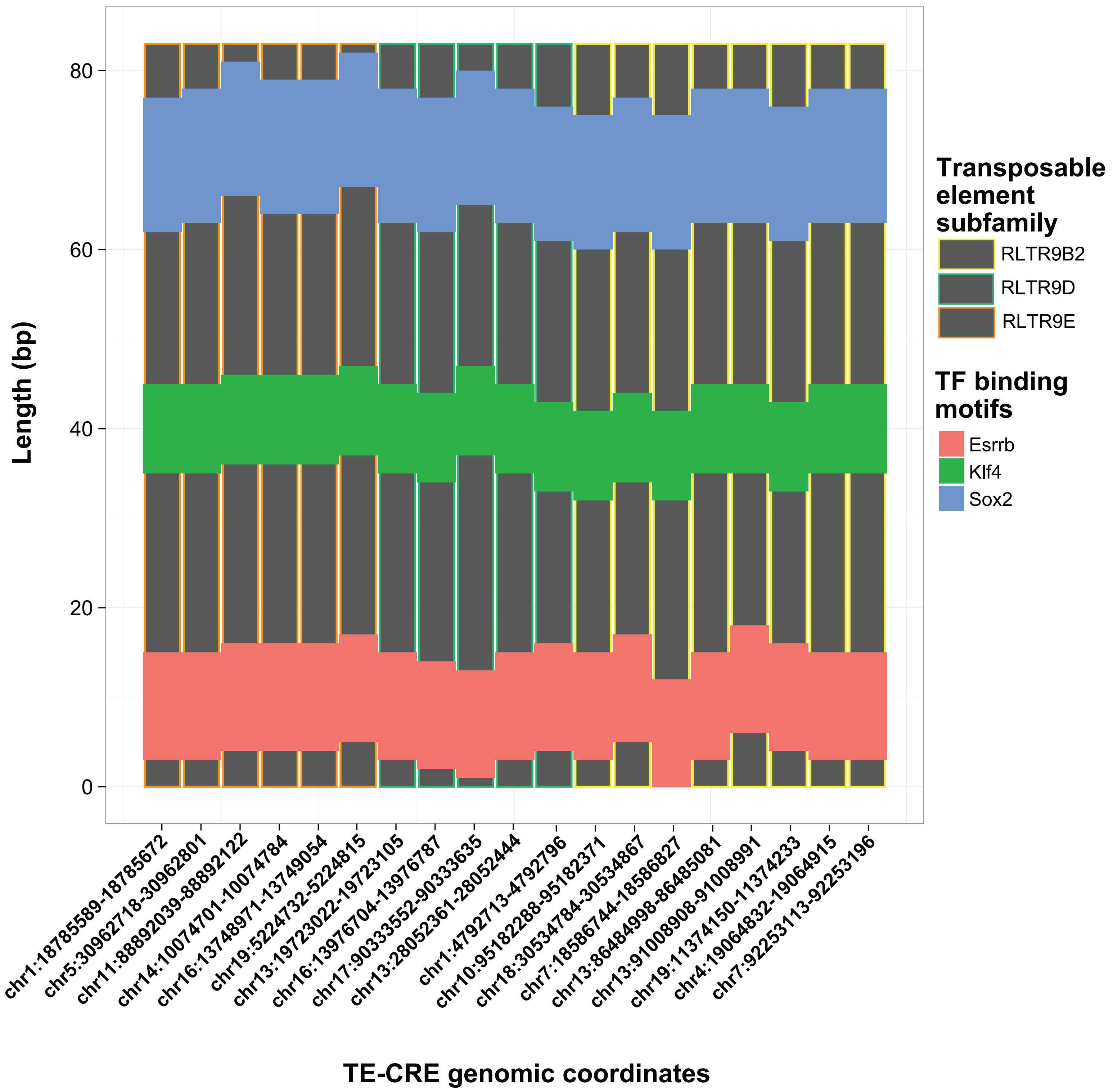

Supplementary Figure 12: Motif-annotations in TE cis-regulatory elements used in CRE-seq (Kwasnieski J, PNAS, 2012). We tested the effect of the Esrrb, Klf4, and Sox2 (EKS) motifs in these elements by mutating individual motifs and all three motifs.

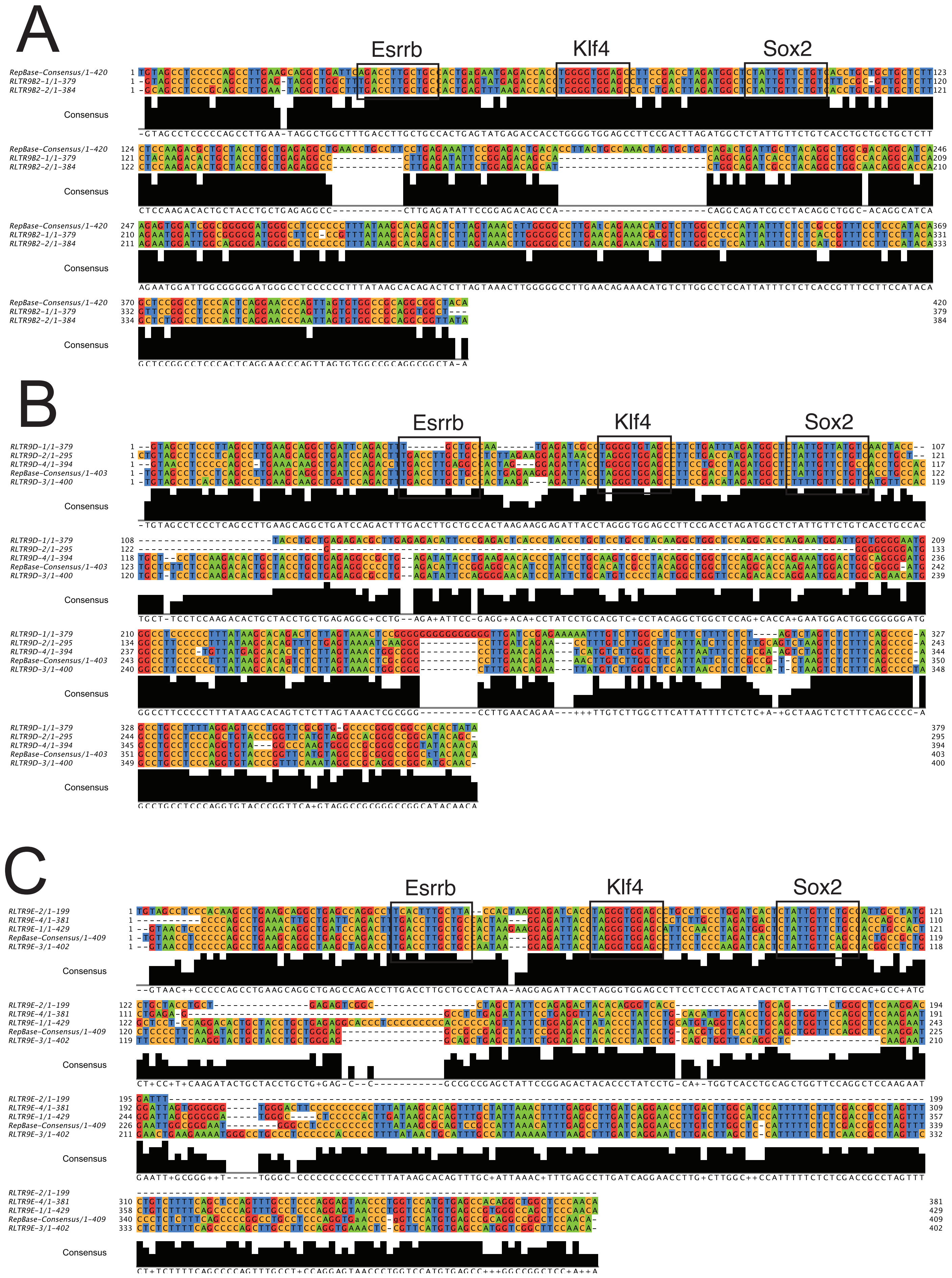

Supplementary Figure 13: Comparison between the RepBase-consensus and genomic TE sequences for TE subfamilies - (A) RLTR9B2, (B) RLTR9D, and (C) RLTR9E. Each row represents the genomic and RepBase-consensus sequence of each TE subfamily. The "Consensus" panel represents the most-frequent nucleotide in a position, based on the multiple-sequence alignment (created by ClustalO (65)).

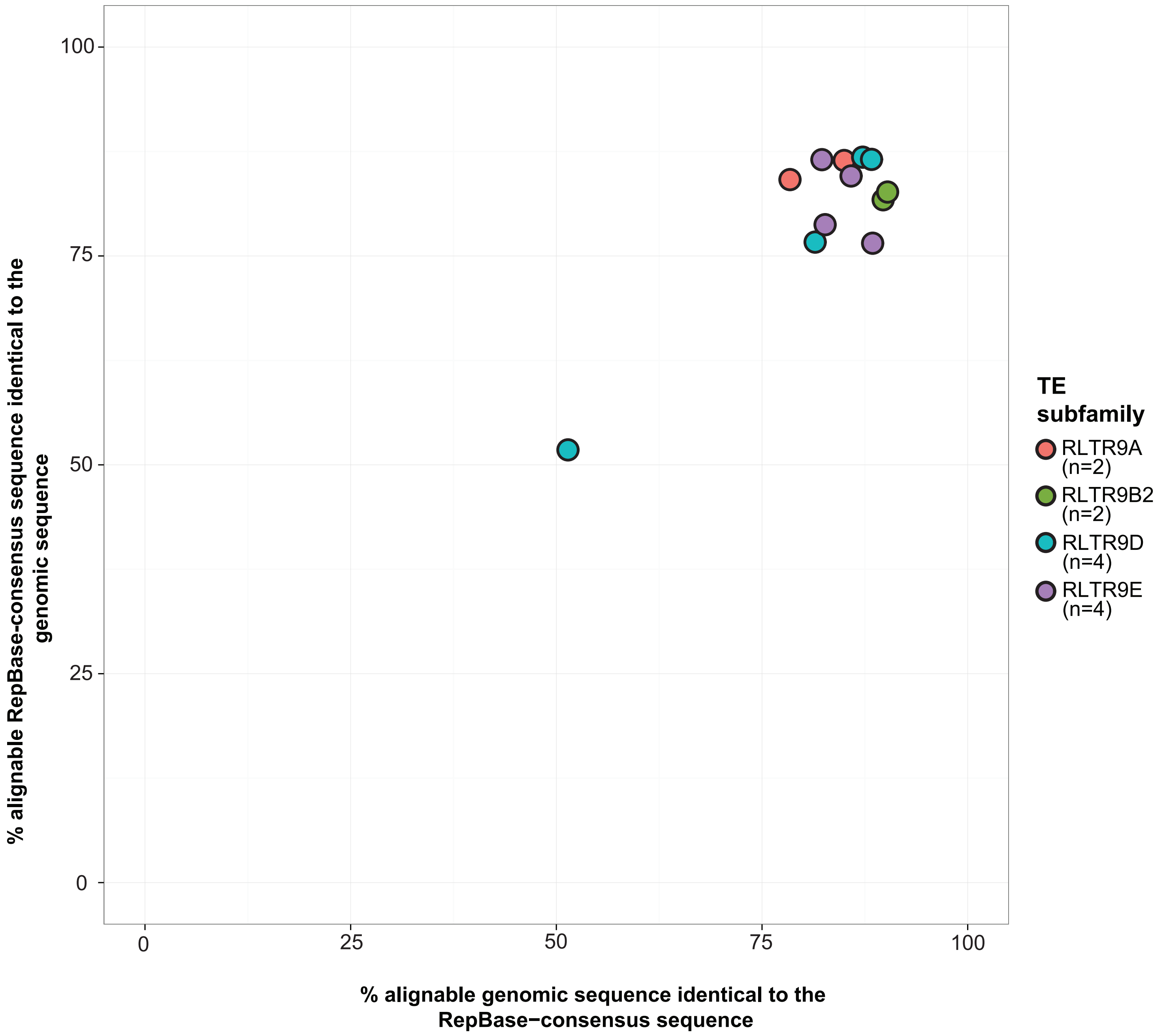

Supplementary Figure 14: Comparison between sequence identity between the RepBase-consensus (x-axis) and genomic TE (y-axis) sequences for TE subfamilies - RLTR9A, RLTR9B2, RLTR9D, and RLTR9E. Each dot represents the pairwise alignment between the RepBase-consensus sequence and the genomic copy of four TE subfamilies (i.e., RLTR9A, RLTR9B2, RLTR9D, and RLTR9E) that we characterized in reporter assays. In total, we tested 12 genomic copies belonging to these four TE subfamilies (number of experimentally tested genomic copies represented by 'n' in the legend), and there is one dot per genomic copy. Each dot represents the percentage of the RepBase-consensus (on the x-axis) and genomic (on the y-axis) sequence that is identical based on a pairwise alignment using *blast2* (Altschul SF, et. al., J. Mol. Biol., 1990).

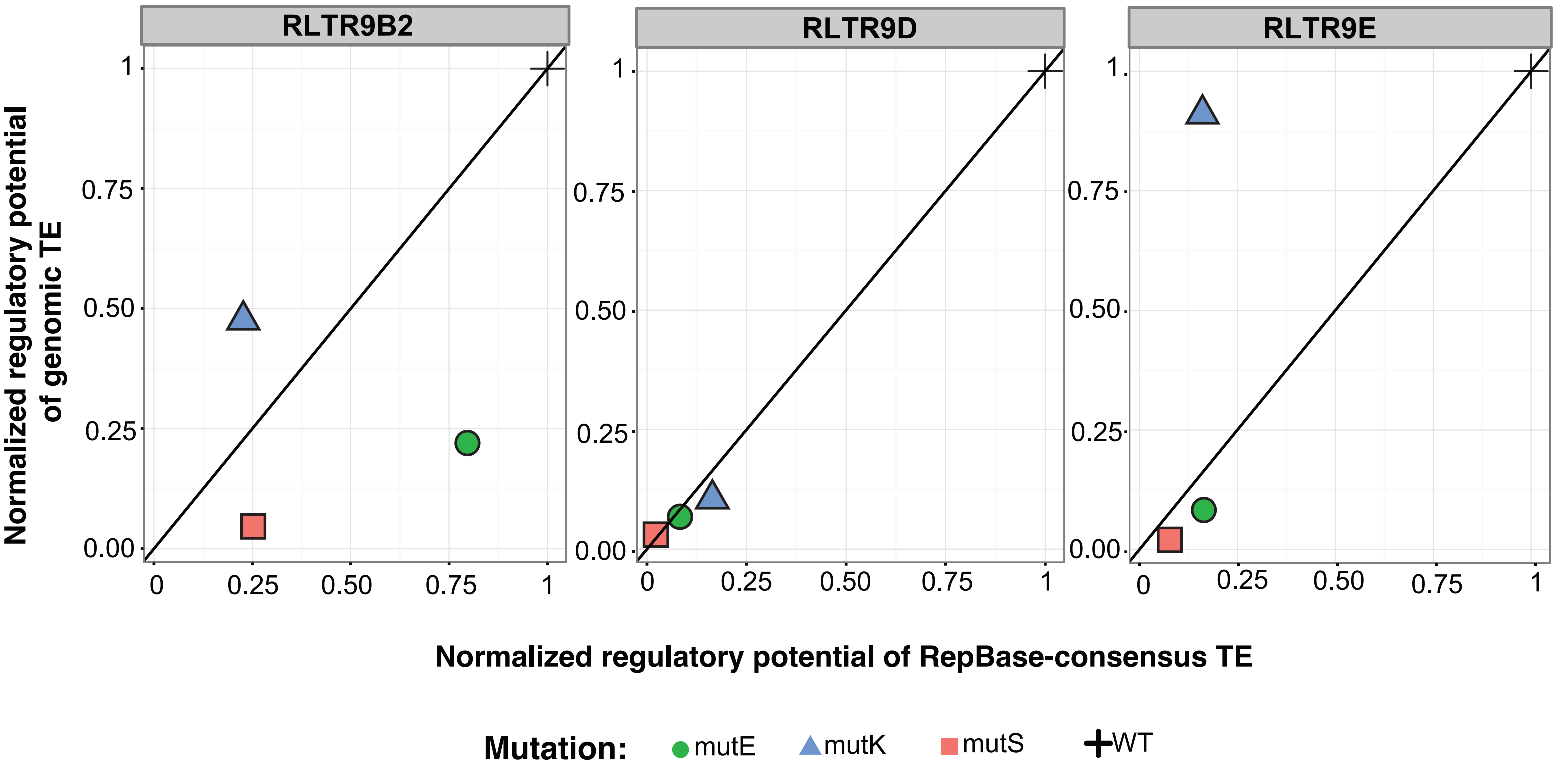

Supplementary Figure 15: Comparing the effect of mutations to the EKS motif-module in genomic TE sequences and the respective RepBase-TE-consensus sequence. We compared the normalized regulatory potential of the RepBase-consensus TE (x-axis) and genomic TE (y-axis). Each point represents a different TF motif that was mutated (legend describes the shape and color associated with each mutation). We compared the regulatory potential of the mutant sequences normalized to its respective wildtype sequence.

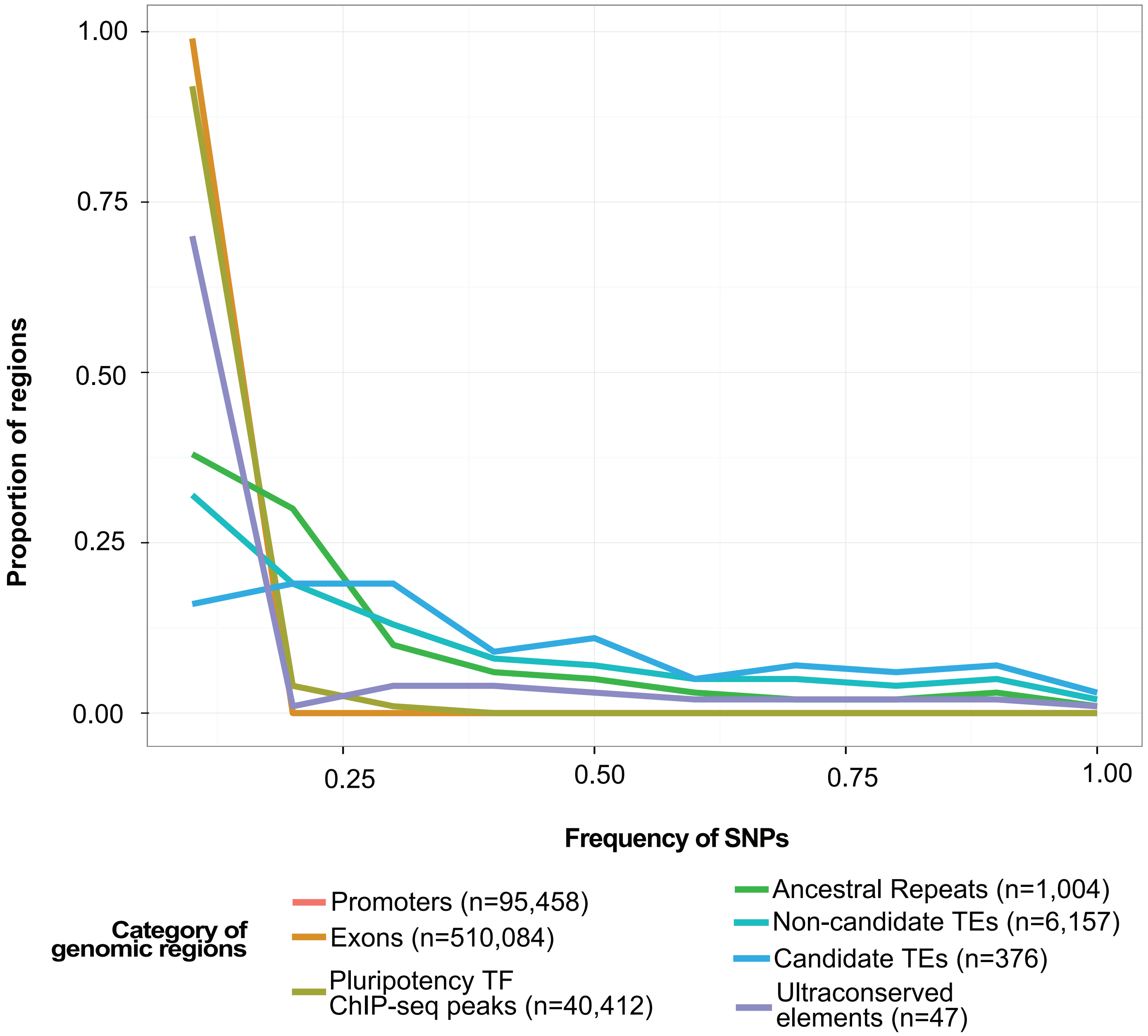

Supplementary Figure 16: Proportion of genomic regions (y-axis) associated with SNP frequency (x-axis) for mouse promoters, exons, ChIP-seq binding peaks used in this study (non-TE overlapping), ancestral repeats, TEs bound by multiple TFs (i.e., Candidate TEs), TEs bound by one TF and unbound TEs (i.e., Non-candidate TEs), and ultraconserved elements. In the legend, 'n' in the parentheses represents the number of genomic regions considered. We used SNPs identified from the mouse genome sequencing project (Keanne, TM, et al., Nature 2011; Nikolskiy, I, et al., BMC Genomics, 2015). Similar to the standard evolutoinary analysis metric of derived allele frequency (DAF), we used SNPs in nineteen mouse strains (from the two referenced studies) to determine the frequency of SNPs in the TEs and other control regions. For comparison, we used genetic elements that are known to be under purifying selection pressures (i.e., promoters, exons, and ultraconserved elements (Pennachio, LA, et al., Nature 2006), and neutrally evolving sequences (i.e., ancestral repeats defined as human-mouse orthologous TEs). The candidate TEs (six TE subfamilies that are bound >= 2 pluripotency TFs) have a similar distribution of SNP frequency scores as neutrally evolving sequences.

Supplementary Table 1: Percentage of pluripotency TF *in vivo* binding peaks in TEs.

Transcription Factor (TF)	Total number of peaks in the genome	Number of peaks in TEs	Percentage of peaks in TEs
Esrrb	21644	5719	26.423
Klf4	10872	1449	13.328
Nanog	10342	2946	28.486
Oct4	3761	760	20.207
Sox2	4525	1218	26.917

Supplementary Table 2: Percentage of clusters of TF binding peaks in TEs.

Number of peaks in the cluster	Total number of peaks in the genome	Number of peaks in TEs	Percentage of peaks in TEs (%)
1	35765	8005	22.382
2	4377	921	21.041
3	1437	299	20.807
4	449	96	19.238
5	103	15	14.563

Supplementary Table 3: TE subfamilies that showed statistical significance (hypergeometric p-value < $1e^{-5}$) in their enrichment for clusters of *in vivo* TF binding sites

Number of TFs	TE Subfamily	TE Class	TE Family	Number of TEs overlapping TF-clusters	Log-Odds Ratio	p-value
1	ID_B1	SINE	B4	1945	3.368	0
1	RLTR13D1	LTR	ERVK	39	2.632	3.43E-24
1	RLTR13D6	LTR	ERVK	143	3.814	3.91E-132
1	RLTR23	LTR	ERV1	48	2.207	1.15E-09
1	RLTR25A	LTR	ERVK	60	2.008	1.85E-14
1	RLTR25B	LTR	ERVK	91	2.541	2.74E-23
1	RLTR41	LTR	ERV1	62	3.042	1.39E-26
1	RLTR9A2	LTR	ERVK	16	4.063	1.93E-12
1	RLTR9A	LTR	ERVK	87	3.683	8.78E-50
1	RLTR9B2	LTR	ERVK	51	5.141	3.05E-54
1	RLTR9D	LTR	ERVK	96	4.778	1.44E-89
1	RLTR9E	LTR	ERVK	97	3.761	2.93E-63
1	RMER10A	LTR	ERVL	86	2.446	6.40E-21
1	RMER10B	LTR	ERVL	42	2.054	2.67E-06
1	RMER16	LTR	ERVK	60	2.874	7.09E-24
2	RLTR11B	LTR	ERVK	17	4.187	2.40E-13
2	RLTR12B	LTR	ERVK	15	3.358	5.94E-09

2	RLTR13D6	LTR	ERVK	82	6.043	1.10E-129
2	RLTR25A	LTR	ERVK	12	2.716	4.55E-06
2	RLTR25B	LTR	ERVK	19	3.312	1.38E-09
2	RLTR9A	LTR	ERVK	27	5.026	1.40E-26
2	RLTR9B2	LTR	ERVK	25	7.143	8.01E-42
2	RLTR9D	LTR	ERVK	31	6.178	2.71E-42
2	RLTR9E	LTR	ERVK	46	5.715	2.02E-56
2	RMER10A	LTR	ERVL	21	3.443	2.71E-11
2	RMER16	LTR	ERVK	23	4.522	3.09E-20
3	RLTR9A	LTR	ERVK	12	5.462	3.68E-14
3	RLTR9B2	LTR	ERVK	15	8.013	1.58E-29
3	RLTR9D	LTR	ERVK	12	6.416	4.23E-18
3	RLTR9E	LTR	ERVK	48	7.383	8.46E-83
4	RLTR9D	LTR	ERVK	12	8.094	3.77E-24
4	RLTR9E	LTR	ERVK	20	7.799	6.58E-38

Supplementary Table 4: Expected (labeled "Exp") number of genomic regions with multiple TF binding sites for candidate TE subfamilies, which showed high enrichment of TF binding. Comparing the observed (labeled "Obs") number of regions with binding with the number of expected (labeled "Exp") number of regions.

	**Number of TFs in genomic region**	**1**		**2**		**3**		**4**		**5**	
**TE subfamily**	**Total length in genome (kb)**	**Exp**	**Obs**	**Exp**	**Obs**	**Exp**	**Obs**	**Exp**	**Obs**	**Exp**	**Obs**
**RLTR9A**	519.982	5	82	1	29	0	13	0	3	0	0
**RLTR9B2**	110.93	1	46	0	30	0	15	0	3	0	1
**RLTR9D**	268.543	3	93	0	31	0	14	0	13	0	0
**RLTR9E**	549.178	6	90	1	49	0	52	0	20	0	0
**RLTR13D1**	482.859	5	33	1	6	0	1	0	0	0	0
**RLTR13D6**	780.388	8	129	1	85	0	11	0	0	0	0

Supplementary Table 5A: Primers for targeting TEs from the genome, used in Figure 4A. The TE identifiers listed below are collected into two categories in Figure 4, and are labeled "RLTR9s" and "RLTR13s".

**TE Identifier**	**Left Primer**	**Right Primer**
RLTR9A-1	AGGTCCGGTACCAGTCCTAGGTTATGCCCTTC	GAACTTGCTAGCAGTTACCCAGTGTAGCTGTC
RLTR9A-2	AGGTCCGGTACCCACACGAGACTGTGCCCTTC	GAACTTAGATCTAGCTGGAACCCCTGAAAGGG
RLTR9B2-1	AGGTCCGGTACCCATGAGAAATTGTAGCCTCC	GAACTTAGATCTACTTCTTGTAAGCCACCTGC
RLTR9B2-2	AGGTCCGGTACCGATGGGGAATTGCAGCCTCC	GAACTTAGATCTCAATATTCCCTATAACCGCC

RLTR9D-1	ATTCAAGGTACCCCACCCTATATGTAG CCTCC	GAACTTAGATCTGGACTCTGTCTATAGT GTGGCCGCC
RLTR9D-2	AGGTCCGGTACCCACTGTTGCGTGCTG TATGC	GAACTTAGATCTATCTCACAACTGTAGC CTCC
RLTR9D-3	AGGTCCGGTACCGGCTGTGAATTGTTG CATGC	GAACTTAGATCTGACAATTCAATGTAGC CTCA
RLTR9D-4	AGGTCCGGTACCTGCCATAAGCTGTAA CCT	GAACTTAGATCTTGTAGGTTATTGTTGT ATACCG
RLTR9E-1	AGGTCCGGTACCAGGGGGAAATTGTA ACCTCC	GAACTTAGATCTCATTCTCTGCTGTTGG AAGC
RLTR9E-2	AGGTCCGGTACCCATTCTGTATTGTAA CTCCC	GAACTTAGATCTGTTTACAATATGTTGG GAGC
RLTR9E-3	AGGTCCGGTACCAAATTGGCAGCAAAT CGTCC	GAACTTAGATCTGGTCTTTATGTGTAGC CTCC
RLTR9E-4	AGGTCCGGTACCCCCCCAGCCTGAAA CTTG	GAACTTAGATCTTTTTTACCTGTGTTGG GAGC
RLTR13D6-1	AGGTCCGGTACCAAAATCAACATACTC TCCTT	GAACTTAGATCTTTATGGAAAACCCGTA TCTG
RLTR13D6-2	ATTCAAGGTACCGCCTACCAAGCAATG CAGTCTGCTT	GAACTTAGATCTGCAGAATATGTGCCAC AGGCTGGGC
RLTR13D6-3	AGGTCCGGTACCTTCCTTCCTTGCTAC GTCCA	GAACTTGCTAGCAGGGAGGAAGTGCC ACGGAT
RLTR13D6-4	ATTCAAGGTACCCTAGTTGCCGTAGAC CCTCTGGGTC	GAACTTGCTAGCAAACACTAGCTGCCA CGCCCTGCCC
RLTR13D6-5	AGGTCCGGTACCAAACAAACAACCAG GAGAAC	GAACTTGCTAGCCTCTCTCTTTTGCTACA CCC
RLTR13D6-6	AGGTCCGGTACCCGTGTATGTGTGCTA CATCC	GAACTTGCTAGCAAATAAATAAGAAAG CCAGG
RLTR13D6-7	ATTCAAGGTACCGTTCCTCTGCTGCTAT GCAC	GAACTTAGATCTCTTTGCAGAGTGCTGC AGACCCTTT
RLTR13D6-8	ATTCAAGGTACCTTTTAAATTATGCCAT AGACCAGCC	GAACTTAGATCTAAAGTAATTTTGCTAC ATCCACTCC
RLTR13D1-1	AGGTCCGGTACCCAGTATAGCCTGCTA TACCT	GAACTTAGATCTCTAGGGTAGATGCTAT GCAT
RLTR13D1-2	ATTCAAGGTACCGATATAACCTTGCCA CACCC	GAACTTAGATCTATTCAGGTTATGCCGC AGACCCTCT

Supplementary Table 5B: Genomic coordinates for positive controls, i.e., non-TE genomic regions bound by multiple pluripotency TFs.

Enhancer ID	Nearby Gene	Genomic coordinates	Forward primer	Reverse primer
1	Oct4	chr17:35640880-35641280	TTAGCTAGCTGGAGA GTGCTGTCTAGG	ATAAGCTTTCCAAG TCATCCCCCAGG
2	Nanog	chr6:122657250-122657650	TATGCTAGCTCC CCC ACTTGACCTGAAA	GTAAGCTTCCACA GAAAGAGCAAGAC ACCA
3	Nanog	chr6:122652800-122653200	GAGCTAGCTCTCAAA GACACTAAAGAGGCA	AGTAAGCTTAGAT GCCACCAGGGAAC
4	Stat3	chr11:100800830-100801250	GTGCTAGCACGCCTC CATGCATAATTAA	CGAAGCTTCTGATT CCCACGTGGTAA

5*	Sox2	chr3:34656690-34657130	TTAGCTAGCAATGACT AAGAGTTTCTCCA	AGAAGCTTTTAAAG TCTCCTTCACAAGA

* This genomic region was selected from a previous publication (Li et al., PLoS ONE 2014, doi:10.1371/journal.pone.0114485) that studied an enhancer and near the Sox2 gene.

Supplementary Table 5C: Genomic coordinates for negative controls, i.e. TEs from the same TE subfamilies that are not bound by any of the five pluripotency TFs.

TE-ID	Genomic coordinates	Forward primer	Reverse primer
RLTR9A-2	chr17:69912769-69913057	ATCTAGCTAGCGTTTT ACAACTTGGCTGGAG	ATCCAAGCTTTGTAGC TGTCCAGCAAC
RLTR9A-3	chr6:131950772-131951050	CTGCTAGCTTGTTTGC CACAGTCTGT	CGAAGCTTTGGTCATA TGTAGCTACCAC
RLTR9B2-1	chr9:35857019-35857453	TATGCTAGCAGGGAG GAATTGTAGCCT	TGAAGCTTGTGTAAGC TGTGACCACC
RLTR9B2-2	chr15:46685298-46685715	GATGCTAGCTAGGTA TGGGTGTAGCCT	GTAAGCTTATATGAAG CCGCCGAGG
RLTR9B2-3	chr9:3876631-3877013	TAGCTAGCAACCTCC CTCAGCTTTGAAG	TGAAGCTTACCACCTG TAACTGCCTG
RLTR9D-4	chr13:51700207-51700525	GTCGCTAGCGACAAA AATCTGTAGCCTC	TCGAAGCTTCTGGGG ATTTTTGTAGC
RLTR9D-5	chr4:61781034-61781409	GTCGCTAGCCTAATA AACTGTAGCCTCC	GTAAGCTTGGTATTTC TGGAAGGCAGG
RLTR9D-6	chr19:61262917-61263304	AATGCTAGCCACCAT ATTTTGTAGCCTCCC	CAGAAGCTTATATTGT AACTGTAAGCCACCCG
RLTR9D-8	chr7:93238995-93239268	TGGCTAGCTATAATG CTGTAGCCTCC	TGCAAGCTTCCAATCT GTTGTAGTAAGC
RLTR9E-1	chr11:39621930-39622324	ATCTAGCTAGCTGTAA CCTCCCCAGATC	GTACCAAGCTTCACTG TGTTGGAAACCC
RLTR9E-3	chr8:5490592-5490910	GTCTAGCTAGCTGTA ATCTTCCCAGCCTT	TACCAAGCTTCAGGGT TTACCTGAAAGGG
RLTR9E-4	chr2:134325117-134325493	ATCTAGCTAGCTGACT CTTTGTGGCCTTC	TTACAAGCTTAAGAGC TGCAGCCATCCA
RLTR9E-6	chrX:33090390-33090747	TACTAGCTAGCGGAA AATGTGTAACCTCCC	TACCCAAGCTTTTTGC CAAATTGTTGAGGC
RLTR13D6-2	chr15:45939533-45940436	ATCTAGCTAGCTGCTA CATCCACTCCAGT	ATCCCAAGCTTTGCCA CAGACTAAACTCT
RLTR13D6-3	chr3:157955259-157956103	CTAGCTAGCGCTACA TCCACTCCAGTG	ATTAAAGCTTAAGGTG CAGCTGCCATGG
RLTR13D6-4	chr10:129263868-129264692	ATCTAGCTAGCAGTCT GCTTGGTAGACCTA	ATCCCAAGCTTTGCCA CAGTCTAACCTTTA
RLTR13D6-5	chrX:23517523-23518460	ATCTAGCTAGCTGCTA CATCCACTCCAGT	ATCCCAAGCTTTTAGTT TCAATGCCATGGAC
RLTR13D6-6	chrX:123868460-123869422	ATCTAGCTAGCTGCTA CATCCACACCAGTA	TATACAAGCTTGTAATT GCCGCAGACCGA
RLTR13D6-7	chr7:24805782-24806740	GTCTAGCTAGCGGTG AATTATTGCTACATCC	ATACCAAGCTTTTTGC CATGGACTGGTCT
RLTR13D6-9	chr8:19233601-19234552	AGCTAGCTAGCTACT ACATCCACTCCAATAG	TATACAAGCTTTGCTG CAGATGGACCTCA
RLTR13D6-10	chrX:18352105-18353054	ATCTAGCTAGCTGCTA	ATCCCAAGCTTTGCTA

		CATCCACTCCACTA	CAGATAGTCCTCAGT
RLTR13D1-1	chr4:121503035-121504104	CTCTAGCTAGCCTTTT TTTAGTGCTAGGCAC	TACAAGCTTTGCCACA GACCCTCTTGATT
RLTR13D1-3	chr12:31859774-31860726	CTAGCTAGCTGCTAT GCACACTCCAGT	TACAAGCTTAGTGTTG CTGCAGACCGT

Supplementary Table 6A: Motifs in TE sequences that were targeted for site-directed mutagenesis

TE-ID	Genomic coordinates	TF	Relative Motif Start	Motif Score	Motif Strand	Motif
RLTR9A-1	chr4:82903264-82903586(+)	Oct4	151	4.69	+	TTTGCAACATAA
RLTR9B2-1	chr16:13748942-13749321(+)	Esrrb	32	5.77	+	TGACCTTGCT
RLTR9B2-1	chr16:13748942-13749321(+)	Klf4	64	5.65	+	GGGGTGGAGC
RLTR9B2-1	chr16:13748942-13749321(+)	Sox2	92	5.11	-	CTATTGTTCT
RLTR9D-2	chr13:19722847-19723141(-)	Esrrb	40	5.77	+	TGACCTTGCT
RLTR9D-2	chr13:19722847-19723141(-)	Klf4	72	5.92	+	AGGGTGGAGC
RLTR9D-2	chr13:19722847-19723141(-)	Sox2	100	5.11	-	CTATTGTTCT
RLTR9E-2	chr13:86484962-86485391(+)	Esrrb	38	5.77	+	TGACCTTGCT
RLTR9E-2	chr13:86484962-86485391(+)	Klf4	70	5.92	+	AGGGTGGAGC
RLTR9E-2	chr13:86484962-86485391(+)	Sox2	98	5.11	-	CTATTGTTCT
RLTR9E-2	chr13:86484962-86485391(+)	Klf4	163	5.78	-	CCCCCACCCC

Supplementary Table 6B: Primers for mutating TF motifs (Supp. Table 11A) in four genomic TEs, for mutagenesis luciferase assay

TE	Mutant	Forward primer	Reverse primer
RLTR9A-1	mutO	cgctgagcttactttTGGacataatccagct gaaac	gtttcagctggattatgtCCAaaagtaagctcagc g
RLTR9B2-1	mutE1	gtaggctggctttTaGAttgctgccactgagt at	atactcagtggcagcaaTCtAaaagccagccta c
RLTR9B2-1	mutK2	gtatgagaccacctgCAAtAgagccttccg acttag	ctaagtcggaaggctcTaTTGcaggtggtctca tac
RLTR9B2-1	mutS	gcaacgcggaagacagaCcCCtagagcc atctaag	cttagatggctctaGGgGtctgtcttccgcgttgc
RLTR9D-2	mutE1	ctgatccagactttTaGAttgctgcctcttaga agg	ccttctaagaggcagcaaTCtAaaagtctggat cag
RLTR9D-2	mutK1	gaaggagataacctagAAtAgaAccttctg accatgat	atcatggtcagaaggTtcTaTTctaggttatctcc ttc
RLTR9D-2	mutS1	ccagcaggtgacagaaACCtagagccatc atggt	accatgatggctctaGGTttctgtcacctgctgg
RLTR9E-2	mutE1	ctgatccagactttTaGAttgctgccactaag aagg	ccttcttagtggcagcaaTCtAaaagtctggatc ag
RLTR9E-2	mutK1	agaaggagattacctagAAtAgagcattcc aacctagatg	catctaggttggaatgctcTaTTctaggtaatctc cttct
RLTR9E-2	mutS1	gtggcaggtggcagaaACCtagagccatct aggt	acctagatggctctaGGTttctgccacctgcca c
RLTR9E-2	mutK2	tccagaataactgggCAAtgggAgggggga gggtg	caccctcccccTcccaTTGcccagttattctgg a

Supplementary Table 7: Genomic coordinates of TEs cis-regulatory elements (CREs) that were selected and tested using the CRE-seq assay.

TE-CRE type	TE subfamily	Genomic coordinates of TE-CRE
EKS motif-module	RLTR9B2	chr19:18785589-18785672
	RLTR9B2	chr5:30962718-30962801
	RLTR9B2	chr11:88892039-88892122
	RLTR9B2	chr14:10074701-10074784
	RLTR9B2	chr16:13748971-13749054
	RLTR9B2	chr19:5224732-5224815
	RLTR9D	chr13:19723022-19723105
	RLTR9D	chr16:13976704-13976787
	RLTR9D	chr17:90333552-90333635
	RLTR9D	chr13:28052361-28052444
	RLTR9D	chr1:4792713-4792796
	RLTR9E	chr10:95182288-95182371
	RLTR9E	chr18:30534784-30534867
	RLTR9E	chr7:18586744-18586827
	RLTR9E	chr13:86484998-86485081
	RLTR9E	chr13:91008908-91008991
	RLTR9E	chr19:11374150-11374233
	RLTR9E	chr4:19064832-19064915
	RLTR9E	chr7:92253113-92253196
Negative control	RLTR9B2	chr10:51536510-51536593
	RLTR9B2	chr1:101530178-101530261
	RLTR9B2	chr17:74402694-74402777
	RLTR9B2	chr3:137937614-137937697
	RLTR9D	chr10:114651926-114652009
	RLTR9D	chr10:14588925-14589008
	RLTR9D	chr12:120319115-120319198
	RLTR9D	chr8:3890885-3890968
	RLTR9E	chr10:129442561-129442644
	RLTR9E	chr1:10161277-10161360
	RLTR9E	chr1:123645300-123645383
	RLTR9E	chr7:111161864-111161947
	RLTR9E	chr9:100493278-100493361

Supplementary Table 8: Mutations in EKS motifs in TE-CREs

Transcription Factor	Wild type motif	Mutated motif

Esrrb	GCAGCAAGGTCA	GCAGCAGTCTCA
Klf4	AGGGTGGAGC	ACAATGGAGC
Sox2	CTATTGTTCTGTCAC	CTAGGTTTCTGCCAC

Supplementary Table 9: RepBase-consensus sequences synthesized. Nucleotides in lowercase represents positions where the RepBase-consensus was ‘N’ and we replaced it with the most frequent nucleotide at that position from the genomic TEs of that subfamily

TE Subfamily	RepBase-consensus sequence
RLTR9A	TGTGCCCTTCCCCCAGCCTTGAGCAGGCTGAATCAGACCTTGTTTTTGCCACAGTCGG<br>CTAGCCCACCTAGGGTGGAGTCTTCCGACCTAGGTAAAGTCTATTGTCCTGCCACATCT<br>GCAGCTTCCTGCAAGAAGCCACTACCTGCTGAGCAGCTGAGCTTGTTTTCCTGACGAG<br>ATCCTGACAAAGCAAGAGATCCTGGCATAGTGGGGGATGGGTTCCCCCCCCTTTAAA<br>GTTCAGACTCAAGAATTAAACATTGAGCCTTGATCAGAGAACTTTGTCTTGGCTCGTTA<br>TTTCTCTCGCCGTCCTTCCCATCCATCCCCAGCTTTCCTTTCAGGAACCCAGTAACCCGT<br>GGCTGCTGGCGGCTACA
RLTR9B2	TGTAGCCTCCCCCAGCCTTGAAGCAGGCTGATTCAGACCTTGCTGCCACTGAGAATGA<br>GACCACCTGGGGTGGAGCCTTCCGACCTAGATGGCTCTATTGTTCTGTCACCTGCTGC<br>TGCTCTTCTCCAAGACGCTGCTACCTGCTGAGAGGCTGAACCTGCCTTCCTGAGAAATT<br>CCGGAGACTGACACCTTACTGCCAAACTAGTGCTGTCAGACTGATTGCTTACAGGCTG<br>GCGACAGGCATCAAGAGTGGATCGGCGGGGGATGGGCCTCCCCCCTTTATAAGCAC<br>AGACTCTTAGTAAACTTTGGGGCCTTGATCAGAAACATGTCTTGGCCTCCATTATTTCTC<br>TCGCCGTTTCCTCCCATACAGCTCCGGCCTCCCACTCAGGAACCCAGTTAGTGTGGCC<br>GCAGGCGGCTACA
RLTR9D	TGTAGCCTCCCTCAGCCTTGAAGCAGGCTGATCCAGACTTTGACCTTGCTGCCACTGA<br>GAAGGAGATTACCTAGGGTGGAGCCTTCCGACCTAGATGGCTCTATTGTTCTGTCACC<br>TGCCACTGCTCTTCTCCAAGACACTGCTACCTGCTGAGAGGCCCCTGAGACATTCCGG<br>AGGCACATCCTATCCTGCACATCGCCTACAGGCTGGCTCCAGGCACCAAGAATGGACT<br>GGCGGGGATGGGCCTTCCCCCTTTATAAGCACAGTCTCTTAGTAAACTCGCGGGCCTT<br>GAACAGAAAACTTGTCTTGGCTTCATTATTCTCTCGCCGTCTAAGTCTCTTTCAGCCCCA<br>GCCTGCCTCCCAGGTGTACCCGGTTCATGTAGGCCGCGGGCCGGCTTACAACA
RLTR9E	TGTAACCTCCCCCAGCCTGAAGCAGGCTGAGCCAGACCTTGACCTTGCTGCCACTAAG<br>GAGATTACCTAGGGTGGAGCCTTCCTCCCTAGATCACTCTATTGTTCAGCCACTGCCGC<br>TGCTCCCCTTCAAGATACTGCTACCTGCTGGGAGGCCGCCGAGCTATTCCGGAGACTA<br>CACCCTATCCTGCACGTCGTCACCTGCAGCTGGTTCCAGGCTCCAAGGATGAATTGGC<br>GGGAATGGGCCTTCCCCCCCCTTTATAAGCGCAGTCCGCCATTAAACATTTGAGCCTT<br>GATCAGGAACCTTGTCTTGGCTCCATTTTTCTCTCGACCGCCTAGTTTCCCTCTCTTTCA<br>GCCCCGGCCTGCCTCCCAGGTGAACCCGGTCCATGTGAGCCGCAGGCCGGCTCCAA<br>CA

Supplementary Table 10: Motifs in RepBase-consensus sequences

TE Subfamily	Length of RepBase-TE-Consensus (bp)	Motif Start	Motif Stop	Motif Orientation	TF	Motif Score	P-value
RLTR9A	368	70	80	+	Klf4	3.18	-9.68
RLTR9A	368	99	114	+	Sox2	3.71	-10.62
RLTR9B2	420	35	47	-	Esrrb	1.96	-7.59
RLTR9B2	420	67	77	+	Klf4	3.80	-10.66
RLTR9B2	420	95	110	+	Sox2	3.13	-9.34

RLTR9E	409	34	46	-	Esrrb	3.10	-9.34
RLTR9E	409	40	52	-	Esrrb	4.07	-11.15
RLTR9E	409	69	79	+	Klf4	3.81	-10.68
RLTR9E	409	97	112	+	Sox2	2.45	-8.03

Supplementary Table 11: Primers for mutating TF motifs in three RepBase-consensus sequences, for the mutagenesis experiment

TE Subfamily	Mutant	Forward primer	Reverse Primer
RLTR9B2	mutE	tctcagtggcagcaaTCtAtgaatcagcctgcttc	gaagcaggctgattcaTaGAttgctgccactgaga
RLTR9B2	mutK	agaatgagaccacctggAAtAgagccttccgaccta	taggtcggaaggctcTaTTccaggtggtctcattct
RLTR9B2	mutS	gacctagatggctctaGATttctgtcacctgctg	cagcaggtgacagaaATCtagagccatctaggtc
RLTR9D	mutE	ggctgatccagactttTaGAttgctgccactgaga	tctcagtggcagcaaTCtAaaagtctggatcagcc
RLTR9D	mutK	gaaggagattacctagAAtAgagccttccgacc	ggtcggaaggctcTaTTctaggtaatctccttc
RLTR9D	mutS	gacctagatggctctaGATttctgtcacctgccac	gtggcaggtgacagaaATCtagagccatctaggtc
RLTR9E	mutE	gctgagccagaccttTaGAttgctgccactaag	cttagtggcagcaaTCtAaaggtctggctcagc
RLTR9E	mutK	aaggagattacctagAAtggagccttcctcccta	tagggaggaaggctccaTTctaggtaatctcctt
RLTR9E	mutS	cctagatcactctaGATttcagccactgccgct	agcggcagtggctgaaATCtagagtgatctagg

Supplementary Table 12: Primers used for qRT-PCR to quantitate the change in expression level after CRISPR-mediated deletion of RLTR9E in Akap12 intron.

Gene Name	Forward Primer	Reverse Primer
Gapdh	aggtcggtgtgaacggatttg	ggggtcgttgatggcaaca
Akap12	ctacaggagccaaagggaga	tttggtctcaaggtcccaac
Lrp11	ggagccatgggattaaatga	ctcgtctctgggtgggatac
Nup43	cctgcagaccacagagacct	tcaaaccctccgtcagaatc
